# Evaluation of an *in vivo* pulmonary aspergillosis model for triazole susceptibility breakpoint development

**DOI:** 10.1128/aac.01643-25

**Published:** 2026-01-30

**Authors:** Alex Lepak, Sujata M. Bhavnani, Mariana Castanheira, Christopher M. Rubino, Jeffrey P. Hammel, M. Courtney Safir, Justin Massey, William Hartman, Paul Ambrose, David Andes

**Affiliations:** 1 University of Wisconsin 5228, Madison, Wisconsin, USA; 2 Institute for Clinical Pharmacodynamics 537914, Schenectady, New York, USA; 3 Element 138461, North Liberty, Iowa, USA; University Children's Hospital Münster, Münster, Germany

**Keywords:** breakpoint, PK/PD, posaconazole, *Aspergillus*

## Abstract

Use of mouse infection models for antimicrobial pharmacokinetic/pharmacodynamic (PK/PD) analysis can assist in dosing regimen design and susceptibility breakpoint development. A major hurdle for clinical translation of *in vivo* study output is defining the model endpoint linked to clinical success. Validation of the *in vivo* endpoint requires a clinical data set composed of success or failure linked to minimum inhibitory concentration (MIC), dosing regimen, and if possible human pharmacokinetic measures. The present studies utilized a clinical library of eight *Aspergillus fumigatus* strains in a mouse pneumonia model to define the endpoint associated with humanized treatment regimens of the triazole, posaconazole. This includes wild-type strains associated with successful treatment and strains with resistance mutations leading to elevated MICs and associated with treatment failure. We found humanized posaconazole exposures resulted in a net stasis or net decrease in organism burden in the animal model compared to the start of therapy for all wild-type strains. However, a net increase in organism burden despite treatment with the humanized regimen was noted for strains with higher MIC values and defined Cyp51 mutations. The ratio of posaconazole free-drug area under the concentration-time curve to the MIC (AUC/MIC) associated with a stasis endpoint in the mouse model was then utilized with *in vitro* surveillance data and a human posaconazole population pharmacokinetic model to perform simulations and PK/PD target attainment analyses. The results of these analyses demonstrated >90% probability of PK/PD target attainment for *A. fumigatus* strains with MICs of ≤0.5 mg/L, thus supporting this susceptible breakpoint threshold.

## INTRODUCTION

Pharmacokinetic/pharmacodynamic (PK/PD) antimicrobial studies in preclinical infection models have been shown useful for predicting outcomes in patients (1–3). Application of these approaches has been used to select dosing regimens for clinical trials and for identification of minimum inhibitory concentration (MIC) thresholds to define susceptibility breakpoints. A key requirement for translation of output from the experimental models is linking the efficacy endpoint in the model to outcome in patients. These determinations require clinical data with drug and dose information as well as an isolate with MIC (4, 5). Studies validating relevant *in vivo* model endpoints have been pioneered and widely utilized with antibacterials and numerous bacterial pathogens for sepsis, pneumonia, sinusitis, otitis media, and soft tissue infection (6). More recently, these approaches have been shown useful with antifungals in the setting of invasive candidiasis (7, 8). Emerging investigations are attempting to apply these tools for other infection scenarios including different infection sites and pathogens. The present studies utilize a humanized regimen of a triazole, posaconazole, in a mouse invasive pulmonary aspergillosis model, including isolate MICs linked to clinical success and failure to identify the endpoint organism burden in the lung against the two groups of strains (9–11). The “validated” stasis endpoint ratio of area under the posaconazole concentration time curve to MIC (AUC/MIC ratio) target was then considered relative to surveillance data MIC distribution and posaconazole population pharmacokinetics using Monte Carlo simulation to provide susceptibility breakpoint guidance.

## RESULTS

### Strain collection and characterization: MIC, CYP51 genotype, and *in vivo* fitness

Eight clinical *Aspergillus fumigatus* isolates were chosen for *in vivo* posaconazole treatment studies. The isolates were chosen from the SENTRY database (12). The collection includes a total of 2,091 non-duplicate *A. fumigatus* isolates collected prospectively from 74 medical centers located in North America (33 sites), Europe (26 sites), the Asia-Pacific region (13 sites), and Latin America (two sites) during 2011 to 2022. These strains were recovered consecutively from patients with bloodstream infections (18 isolates), intra-abdominal infections (one isolate), skin and skin structure infections (109 isolates), urinary tract infections (one isolate), pneumonia in hospitalized patients (1,619 isolates), and 343 isolates were collected from non-specified sites.

The strain selection criteria for *in vivo* study included posaconazole MIC, Cyp51 genotype, and fitness in the mouse lung infection model (Table 1). Susceptibility testing was performed via Clinical and Laboratory Standards Institute (CLSI) M38 microbroth dilution (13). The strain collection MIC range was 0.25–1.0 mg/L, which includes MIC values associated with failure in clinical case reports (9, 10). The Cyp51 genotypes included wild-type as well as common mutants associated with higher triazole MIC values (14–16) (SRA number for these sequences is PRJNA1381729). The fitness of the selected isolates was relatively similar based upon the increase in lung burden in control mouse lungs over the 96-h study period.

**TABLE 1:** Clinical *A. fumigatus* isolates, MIC, Cyp51 genotype, and mouse fitness

*A. fumigatus* isolate	Posaconazole MIC (mg/L)	Cyp51 genotype	Control growth (Log_10_ CE/Lung)
1,070,650	0.25	CYP51 A9T	1.72
1,072,954	0.25	WT	2.21
1,053,216	0.5	CYP51 I242V	2.75
1,072,941	0.5	WT	1.91
1,077,395	1	Cyp51 TR34/L98H	1.92
1,100,802	1	Cyp51 TR34/L98H	1.98
F16216	1	Cyp51 TR34/L98H	2.37
F13747	1	Cyp51 G434C	1.94

### Posaconazole mouse pharmacokinetics, dose response, and pharmacodynamic analysis

A neutropenic, corticosteroid immunosuppressed mouse invasive pulmonary aspergillosis (IPA) model was utilized as previously described using a 10^7^ conidia/mL inoculum (17–19). Aspergillus fungal burden was determined by real-time quantitative PCR (qPCR) using previously reported methods (18–20).

**Fig 1:**
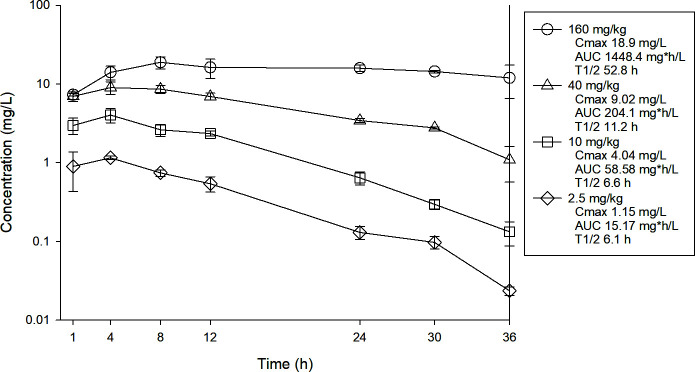
Single-dose plasma concentration-time pharmacokinetic curves for posaconazole. Four different doses of posaconazole that varied by fourfold concentration on mg/kg basis were administered to mice by oral route. Groups of three mice were sampled at each time point. Each symbol represents the mean ± standard deviation for three mice. AUC 0-∞ was calculated by the trapezoidal rule.

Plasma pharmacokinetics were performed in the mouse model to describe the plasma exposure over the dose range utilized in treatment studies and to identify the treatment regimen that approximates the exposure of the posaconazole delayed release tablet 300 mg/day regimen in patients based upon the AUC (37.9 mg*h/L) (11). Single doses of posaconazole were administered by oral gavage at doses of 2.5, 10, 40, and 160 mg/kg. Plasma was obtained from each animal by centrifugation of anticoagulated blood obtained by cardiac puncture into Na-EDTA tubes. Plasma was stored at −70°C until drug assay. Drug concentrations were determined using liquid chromatography-tandem mass spectrometry (LC-MS/MS). The lower limit of quantitation was 0.0025 mg/L. The time course plasma exposures are shown in Fig. 1 (Table S1). The beta-elimination half-life ranged from 6.1 to 52.8 h. The plasma AUC 0h to ∞ (mg*h/L) was linear over the first three dose levels and ranged from 15.17 to 1448 mg*h/L over the 64-fold dose range. Protein binding in mouse serum determined by ultrafiltration methods at concentrations of 1, 5, and 25 mg/L was 99.4% and similar over the concentration range (Table S2). The mouse dose that approximated the human exposure was determined by interpolation to be a dose of 7 mg/kg/day.

**Fig 2:**
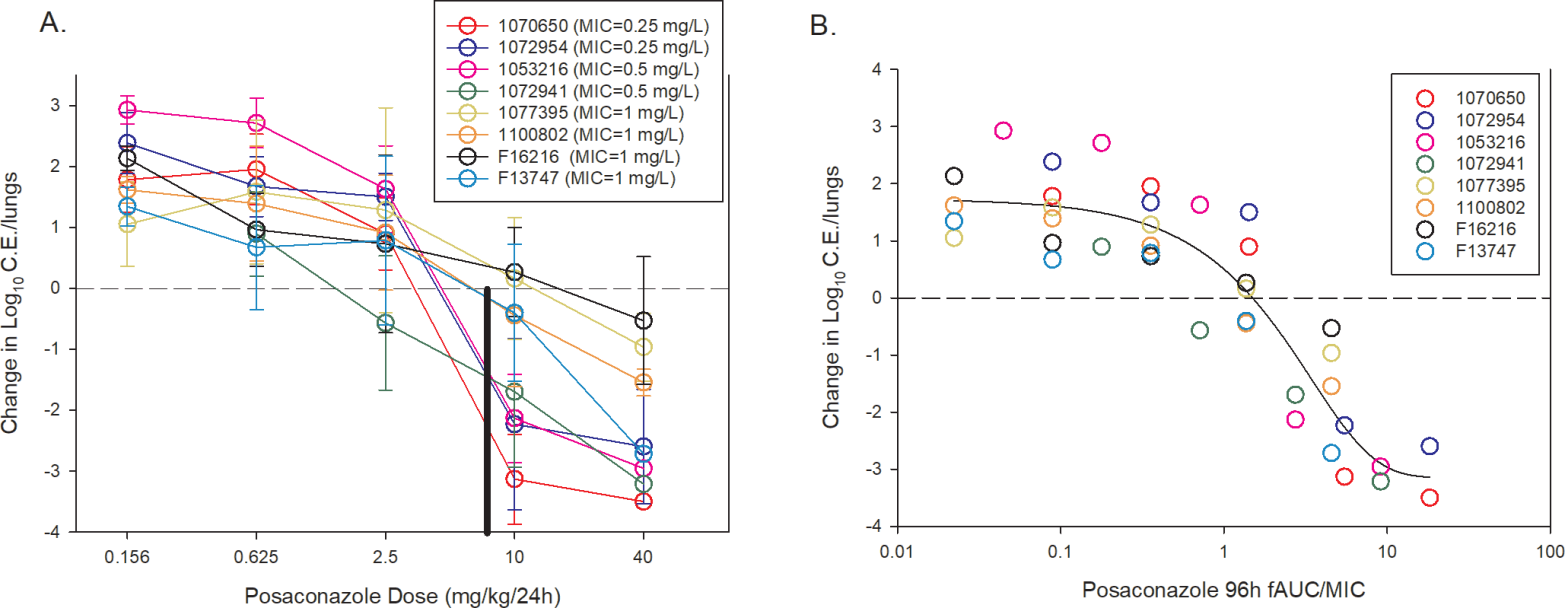
(**A**) Posaconazole dose-response (mg/kg/24 h) curves for each *A. fumigatus* strain. Each symbol is the mean and standard deviation from four treated mice at each dose level. The horizontal dashed line represents net stasis of burden from the start of therapy. Points above the line represent new growth, and those below the line a net decrease in burden. The solid black horizontal line represents the mouse dose that approximates the human exposure of posaconazole delayed release tablet (DRT) at a dose of 300 mg/day. (**B**) PK/PD regression analysis for free drug AUC/MIC. Each symbol is the mean from four mice. The curve is the best-fit line based upon the sigmoid maximum effect (Emax) model. Also shown is the Emax, 50% maximal effect (ED50), slope of the curve (N), and strength of relationship based on coefficient of determination (R^2^).

Treatment studies were initiated 2 h after infection and drug administered daily for 96 h using one of five dose levels (0.156 to 40 mg/kg/day) via oral gavage. The exposure response results included both ineffective (organism growth compared to start of therapy) and effective (no growth or an organism reduction compared to burden at therapy initiation). In general, the dose response curves are shifted to the left for isolates with lower MICs and to the right for those with higher MICs and known Cyp51 target site mutations (Fig. 2A). At the humanized posaconazole exposure level, at least a stasis effect was observed for strains at MICs of 0.5 µg/mL or less. Conversely, the humanized exposure did not achieve the stasis endpoint for isolates with Cyp51 mutations and higher MICs. On this basis, we propose the posaconazole endpoint in this model that is predictive of treatment success in patients is a stasis endpoint or greater.

Regression of the treatment data using the AUC/MIC PK/PD triazole driver was undertaken to identify the magnitude of the dose and PK/PD index associated with net fungal stasis (i.e., suppressing fungal growth compared with the fungal burden at the start of therapy). The posaconazole free-drug AUC/MIC (fAUC/MIC) values were determined for each isolate and fit to a sigmoid Emax dose-response model shown in Fig. 2B. The posaconazole exposure response data fit the model well, with an R^2^ of 0.84. We observed up to a 3-log_10_ reduction in lung organism burden compared with burden at the start of therapy. Considering free drug plasma concentrations, the posaconazole AUC/MIC values associated with the stasis endpoints for the study strains are shown in Table 2. The median posaconazole dose (mg/kg/24 h) needed to produce a static effect was 4.40 ± 4.19. The corresponding median 96-h fAUC/MIC value associated with the stasis endpoint was a value of 1.41 ± 0.58. While a 1-log organism reduction was achieved for a subset of organisms, this endpoint was not achieved for all strains and thus was not considered further (Table S3).

**TABLE 2:** Posaconazole 96-h total- and free-drug plasma AUC/MIC targets associated with net fungal stasis for *A. fumigatus* isolates evaluated in a neutropenic and corticosteroid murine lung infection model

*A. fumigatus* isolate	MIC (mg/L)	Static dose (mg/kg/24 h)	96-h total drug plasma AUC (mg•h/L)	96-h total drug plasma AUC/MIC	96-h free drug plasma AUC/MIC
1,070,650	0.25	3.16	74.35	297.41	1.78
1,072,954	0.25	3.62	84.76	339.05	2.03
1,053,216	0.5	4.33	100.80	201.60	1.21
1,072,941	0.5	1.68	39.85	79.70	0.48
1,077,395	1	12.21	268.01	268.01	1.61
1,100,802	1	5.81	134.04	134.04	0.80
F16216	1	12.86	279.55	279.55	1.68
F13747	1	4.47	103.85	103.85	0.62
Median		4.40	102.32	234.80	1.41
Mean		6.02	135.65	212.90	1.28
Standard deviation		4.19	89.43	97.48	0.58
%CV		69.71	65.92	45.79	0.58
95% CI lower bound		3.11	73.68	145.35	0.87
95% CI upper bound		8.92	197.62	280.45	1.68

### Posaconazole surveillance MIC distribution

The strain collection described above included 2,091 clinical isolates. The MIC distribution and antimicrobial activity of posaconazole are summarized in Table 3. The posaconazole MIC_50_ and MIC_90_ in the strain collection were 0.25 and 0.5 mg/L, respectively. Cyp51A and Cyp51B alterations for *A. fumigatus* isolates displaying non-WT triazole MIC values are listed in Table S4. The CYP51A gene was sequenced in all non-wild-type isolates. Among 109 (5.2% overall) isolates evaluated, 21 isolates harbored the L98H alteration in Cyp51A preceded by a 34 bp tandem repeat (TR) (Table S4). Additionally, three to five of the following alterations F46Y, M172V, N248T, D255E, and E427K were detected among six isolates. The other 22 isolates harbored Cyp51A amino acid changes. Finally, 16 isolates presented Cyp51B amino acid substitutions.

**TABLE 3:** Posaconazole MIC distribution among clinical isolates

Organism/organism group (no. of isolates)	No. and cumulative % of isolates inhibited at MIC (mg/L) of:											MIC_50_	MIC_90_
	≤0.015	0.03	0.06	0.12	0.25	0.5	1	2	4	8	>		
Posaconazole (2,091)	00.0	30.1	211.1	30,215.6	115,170.6	56,697.7	4,599.9	199.9	1 > 99.9	1,100.0		0.25	0.5

### PK data and population PK model development

The publications describing posaconazole population PK models that were considered are listed in Supplemental Results and Table S5. The model described in the publication from Iwasa et al. was evaluated and deemed sufficiently generalizable given inclusion of both healthy and infected individuals and the identification of relevant covariate relationships (21).

Iwasa et al. found a two-compartment model with sequential zero- and first-order absorption process and linear elimination with allometric scaling to provide the best fit to the data (21). Several covariate relationships were determined to be significant: age on clearance (CL), infection status on the volume of the central and peripheral compartments (Vc and Vp, respectively), food on the absorption rate constant (Ka), and formulation, food, and infection status on bioavailability (F). Observed concentration-time data were not provided in the publication; therefore, confirmation of model replication was carried out by regenerating the PK exposure simulation results from the publication (data not shown). The model description in Iwasa et al. was somewhat incomplete as the centering values for age and weight were not provided and the parameterization of F was not clear. Ultimately, a model that assumes a logit transformation for F adequately captured the simulation results from Iwasa et al. and was considered most generalizable.

Table S6 shows the eight publications identified for the qualification of the population PK model. The fit of the Iwasa population PK model to each qualification study is described in the Supplemental Results Fig. S1 to S8). Overall, the Iwasa population PK model, developed using data from Phase 1 and 3 clinical trials for posaconazole, robustly captured the observed concentration-time data from and had PK parameter estimates that were consistent with those in the majority of the identified independent publications. These results support the model as a viable basis for carrying out model-based simulations to evaluate PK/PD target attainment using posaconazole AUC/MIC targets for efficacy against *A. fumigatus*.

Using the population PK model selected for posaconazole, populations of 5,000 simulated patients were generated separately for fasted and fed patients, given that the model contained a relationship between fed status and posaconazole bioavailability. Patient age and body weight were assigned randomly using truncated normal distributions informed by the distributions of these variables reported for 232 patients with invasive fungal infections (22) that participated in the Phase 3 clinical study, SECURE (23). Distributions of posaconazole free-drug plasma AUC among simulated fasted and fed patients were found to be similar, with median AUC values by day differing by no more than 5.6%. Thus, the fasted and fed simulation populations were combined, providing a total of 10,000 simulated patients.

### PK/PD target attainment

**Fig 3:**
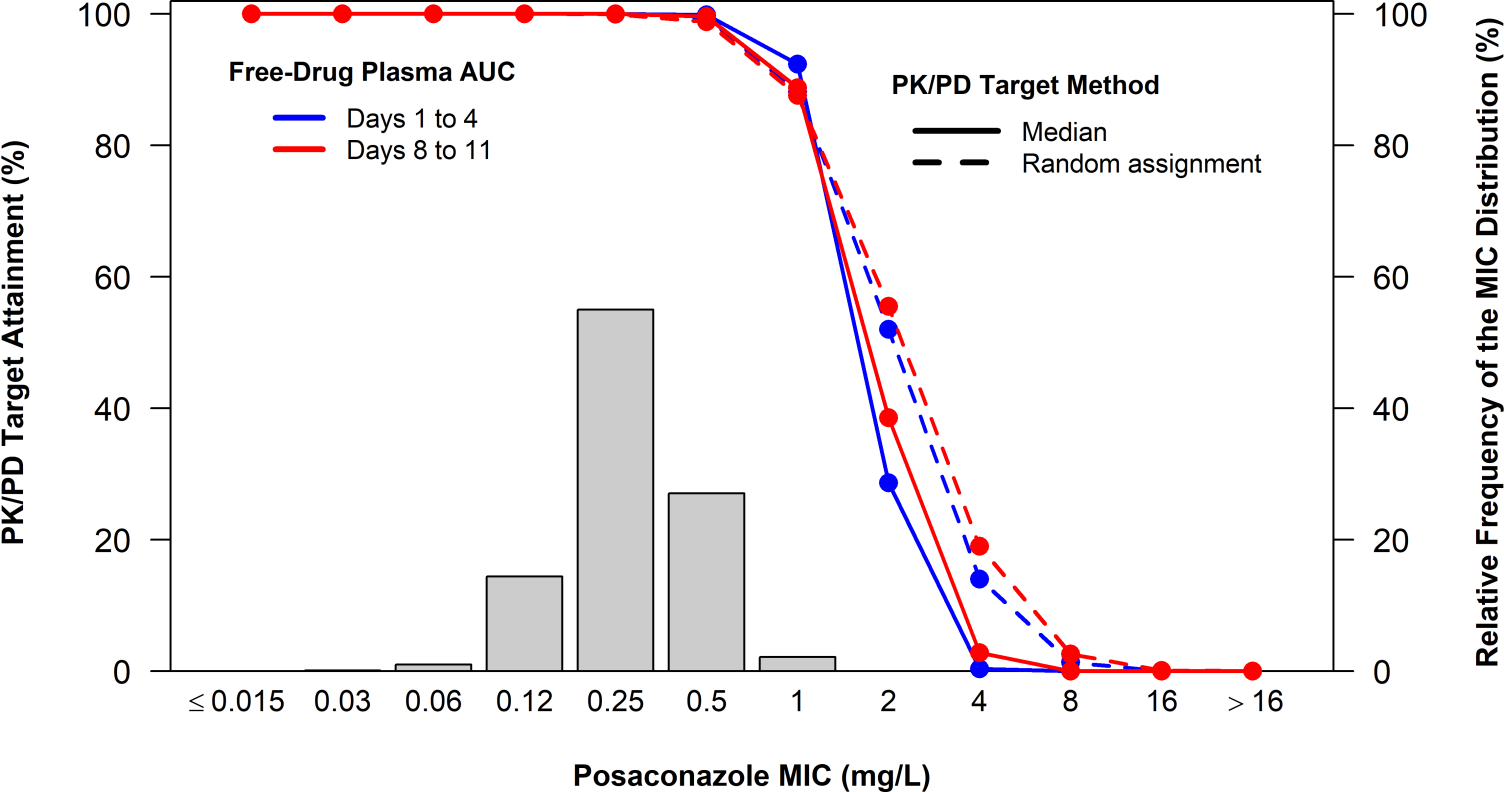
Probability of percent PK/PD target attainment by MIC simulation for posaconazole based upon a net fungal stasis target and a human protein binding estimate of 98.7% among simulated patients after administration of posaconazole 300 mg PO q12h on Day 1 followed by 300 mg PO q24h on Day 2 and thereafter, overlaid upon the posaconazole MIC distribution for 2,091 *A*. *fumigatus* isolates collected from centers worldwide.

Using the average 24-h free drug plasma PK/PD indices calculated for the simulated patients, percent probabilities of attaining median and randomly assigned PK/PD targets for *A. fumigatus* were evaluated. Percent probabilities of PK/PD target attainment by MIC on Days 1 to 4 and Days 8 to 11 based on median and randomly assigned posaconazole free-drug plasma AUC/MIC targets associated with net fungal stasis and a human protein binding estimate of 98.7% among simulated patients after administration of posaconazole 300 mg PO q12h on Day 1 followed by 300 mg PO q24h on Day 2 and thereafter are shown in Table 4. These results, overlaid upon the posaconazole MIC distribution for 2,091 *A*. *fumigatus* isolates collected from centers worldwide, are shown in Fig. 3. Percent probabilities of PK/PD target attainment on Days 1 to 4 and Days 8 to 11 based on randomly assigned free-drug plasma AUC/MIC targets were ≥90% at an MIC value of 0.5 mg/L for all analysis groups. At an MIC value of 1 mg/L, percent probabilities of PK/PD target attainment on Days 1 to 4 and Days 8 to 11 were 88.2 and 87.6%, respectively.

**TABLE 4:** Percent probabilities of PK/PD target attainment by MIC based on median and randomly assigned posaconazole 96-h free drug plasma AUC/MIC targets associated with net fungal stasis for *A. fumigatus* and a human protein binding estimate of 98.7% among simulated patients after administration of posaconazole 300 mg PO q12h on Day 1 followed by 300 mg PO q24h on Day 2 and thereafter^*a*^

MIC (mg/L)	Percent probabilities of PK/PD target attainment by posaconazole MIC			
	Median AUC/MIC target		Randomly assigned AUC/MIC targets	
	Days 1 to 4	Days 8 to 11	Days 1 to 4	Days 8 to 11
0.015	100	100	100	100
0.03	100	100	100	100
0.06	100	100	100	100
0.12	100	100	100	100
0.25	100	100	100	>99.9
0.5	99.9	99.6	99.5	98.8
1	92.3	88.8	88.2	87.6
2	28.7	38.6	52.0	55.5
4	0.36	2.84	14.0	19.0
8	0	0.04	1.35	2.64
16	0	0	0	0.10
32	0	0	0	0

^*a*^ Gray highlight represents acceptable target attainment.

Candidate susceptibility test interpretive criteria (STIC) or susceptibility breakpoint for posaconazole against A. fumigatus were considered based on the results of PK/PD target attainment analyses. Candidate posaconazole-susceptible breakpoints against *A. fumigatus* were based upon the highest MIC value of 0.5 mg/L at which the percent probabilities of PK/PD target attainment were ≥90% based on median and randomly assigned AUC/MIC targets associated with fungal stasis. Percent probabilities of PK/PD target attainment at an MIC value of 1 mg/L ranged from 87.6 to 92.3% across target assignment approaches and time periods. The percentages of *A. fumigatus* isolates inhibited at the candidate posaconazole susceptible breakpoint of 0.5 mg/L and an MIC value of 1 mg/L were 97.7 and 99.9%, respectively. Percent probabilities of PK/PD target attainment at an MIC value of 2 mg/L were ≤55.5%. Based on the results of PK/PD target attainment assessments, posaconazole susceptible, intermediate, and resistant breakpoints of ≤0.5, 1, and ≥ 2 mg/L, respectively, are recommended.

## DISCUSSION

An estimated 6 million patients worldwide are afflicted by invasive fungal infections annually, resulting in nearly 4 million deaths (24, 25). As at-risk immunocompromised populations continue to grow, deaths from fungal infections will continue to rise (26–29). Indeed, fungi are the leading cause of infection-related mortality in most cancer and transplant populations (26, 27, 30, 31). The clinical impact of fungi is so great that the CDC, Infectious Diseases Society of America, and the WHO have placed fungi on the list of top-priority drug-resistant microbes (32–35). Today, only three antifungal drug classes are available for treating invasive disease. The paucity of drug options alone would not be so critical if current antifungal treatments were effective. However, despite therapy, patient survival remains unacceptably low (36–38). For example, the outcomes for *Aspergillus* and other mold pathogens are dismal, with mortality reaching as high as 80% (36, 37, 39). This problem is compounded by the emergence of resistance to first-line antifungal options (15, 16, 40–42). For example, a recent global study of patients with *Aspergillus* pneumonia identified triazole drug resistance in nearly 50% of patients in high-risk groups (15, 43–45).

Antimicrobial PK/PD approaches have improved the rational selection of drug choice, dosing regimen design, and development of susceptibility breakpoints (1, 6). The impact of these PK/PD studies becomes particularly important at times of antimicrobial resistance emergence. These studies utilize a combination of preclinical *in vitro* and *in vivo* infection models that incorporate drug exposure relative to MIC to delineate an exposure linked to success. The model endpoint that defines success in these models requires a clinical validation data set with success and failure linked to MIC and drug exposure. Initial clinical PK/PD studies with antimicrobials accomplished this in the setting of otitis media and emerging resistant *S. pneumoniae* (46). Outcome in the mouse thigh model was linked, and the endpoint in the studies validated based upon benchmarking to human PK/PD exposure response data (47). Subsequent studies have similarly identified translational value of preclinical PKPD for antibacterials in the setting of pneumonia, soft tissue infection, and bacteremia based upon training from clinical data sets (48–51).

Translational application of these tactics to antifungal therapy has lagged use for antibacterials. However, clinical candidemia data sets with failures connected to drug exposure and MIC have provided the opportunity to define a predictive PK/PD endpoint in the neutropenic mouse disseminated candidiasis model. Specifically, pharmacometric analysis of micafungin clinical trials identified an AUC/MIC associated with treatment success (7). The study endpoint in the murine model for this AUC/MIC exposure was a stasis endpoint, or the treatment that eliminated organism growth compared to that present at the start of therapy (52, 53). This observation was useful for the development of echinocandin susceptibility breakpoints for *Candida* species (3, 7, 54). Similar data sets for other fungal species are limited. For invasive aspergillosis, a voriconazole clinical exposure-response relationship was identified due to wide interpatient pharmacokinetic variability observed with this agent (55, 56). Use of this clinical exposure-response data to inform preclinical model endpoints has been further hampered by wide pharmacokinetic variability of voriconazole in rodents, including induction of metabolism over time. Hope et al. utilized another approach, in which a humanized exposure of an approved antifungal (posaconazole) against an isolate of *A. fumigatus* in a preclinical model was used as a benchmark to identify the endpoint and exposure of a novel antifungal, olorofim, that would be expected to lead to clinical success (57). This information was utilized to design olorofim dosing regimens for clinical trials.

The present studies adapted this approach for the study of posaconazole in the neutropenic, corticosteroid-treated model of invasive pulmonary aspergillosis. Specifically, we included humanized posaconazole regimens among the exposure range examined. In addition, we included a study of an *A. fumigatus* strain library that was comprised of isolates with varying MIC and defined Cyp51 resistance mutations. Importantly, the strain collection included isolates with MIC values linked to patient failure from case reports. Posaconazole treatment with the humanized regimen against strains with wild-type Cyp51 genotypes and associated lower MICs resulted in at least a stasis endpoint in the model. Conversely, this regimen did not achieve this model outcome for strains with higher MICs and Cyp51 mutations. We propose a stasis endpoint in this model that would predict clinical efficacy on the basis of these observations.

We subsequently utilized the stasis target from the model, human population pharmacokinetics, and posaconazole MIC distribution and simulation, to evaluate probabilities of PK/PD target attainment by MIC and the percent probability of PK/PD target attainment threshold of ≥90% threshold to guide selection of a susceptibility breakpoint. Given variability in the animal model stasis target among strains, we utilized both the mean and randomly assigned targets in simulations to better account for this expected biological variability. Based on the results of this assessment, we propose a susceptible breakpoint of ≤0.5 mg/L, at which 97.7% of *in vitro* surveillance isolates were inhibited. It should be noted this susceptible breakpoint is consistent with the EUCAST epidemiological cut-off value of 0.5 mg/L for posaconazole (58). However, we note this value is higher than the EUCAST breakpoint for posaconazole and *A. fumigatus* (≤0.125 mg/L) (59). Since percent probabilities of PK/PD target attainment at an MIC value of 1 mg/L approached 90%, 1 mg/L was recommended as an intermediate breakpoint. A resistant breakpoint of ≥2 mg/L was recommended based on percent probabilities of PK/PD target attainment ≤55.5% at an MIC value of 2 mg/L.

We speculate this preclinical model and endpoint will be useful to guide breakpoint development for other drugs in the setting of pulmonary aspergillosis with *A. fumigatus*. We further posit this preclinical model endpoint will be informative in the design of dosing regimens for new agents in development. Future studies could utilize these approaches for other model surrogate endpoints such as serum galactomannan and other emerging fungal pathogens. Continued search for informative clinical data sets will be critical to either validate or refute the model endpoint predictions presented here. Additionally, while the studies presented here utilize population pharmacokinetics that incorporate patient to patient PK variability, we note that therapeutic drug monitoring has been utilized to assist in optimizing triazole exposure (60). Future clinical evaluation of TDM in the setting of these exposure-MIC relationships will also be important.

## MATERIALS AND METHODS

### Fungal identification methods

*A. fumigatus* isolates were cultured and identified using the matrix-assisted laser desorption ionization-time of flight mass spectrometry (MALDI ToF MS) Biotyper (Bruker Daltonics, Billerica, Massachusetts, USA) or DNA sequencing analysis (sequencing of ^28^S and β-tubulin) when an acceptable identification was not achieved by MALDI-TOF MS. Nucleotide sequences were analyzed using Lasergene software (DNAStar, Madison, Wisconsin, USA) and compared with available sequences through the internet using BLAST (https://blast.ncbi.nlm.nih.gov/Blast.cgi).

### CLSI broth microdilution method

All isolates were tested by the broth microdilution method as described by CLSI M38 (2017) document using an automated system to assist with pipetting (13). Frozen-form panels used RPMI 1640 broth supplemented with MOPS (morpholinepropane sulfonic acid buffer) and 0.2% glucose and inoculated with 0.4 to 5 × 10^4^ cells/mL. MIC endpoints were read as 100% inhibition after 48 h of incubation, as recommended by CLSI M38 (2017) (13). The posaconazole concentration range examined was 8–0.015 mg/L.

### Quality control

Quality control (QC) was performed as recommended in the M38 document using the following strains: *Candida parapsilosis* ATCC 22019, *Candida krusei* ATCC 6258, *Aspergillus flavus* ATCC 204304, *A. fumigatus* ATCC 204305, *A. fumigatus* MYA-3626, and *Hamigera insecticola* ATCC MYA-3630. Acceptable MIC ranges for the QC strains were published by CLSI in the M38M51S (2022) document (61).

### Cyp51A and Cyp51B sequencing

*A. fumigatus* isolates exhibiting azole MIC values above the ECV were submitted to whole genome sequencing. Total genomic DNA was used as input material for library construction prepared using the Illumina (San Diego, California, USA) DNA prep library construction protocol following the manufacturer’s instructions. Sequencing was performed on a NextSeq 1000 Sequencer (Illumina). Reads were error-corrected using Sickle version 1.33. Each sample was assembled using a reference-guided assembly in DNASTAR SeqMan NGen v.17.0 (Madison, Wisconsin, USA). DNA regions encoding *cyp51* were compared with the sequences available in the literature, potentially poor source control and/or comorbidities. STIC recommendations in the context of these results and the range of ECOFF values generated were considered. The latter data were evaluated in order to identify STIC that minimized bisection of the wild-type MIC distributions when possible.

### Murine invasive aspergillosis pneumonia model

Six-week-old, specific-pathogen-free, female Institute of Cancer Research/Swiss mice weighing 24–27 g were used for all studies (Harlan Sprague-Dawley, Indianapolis, IN, USA). Animals were maintained in accordance with the criteria of the Association for Assessment and Accreditation of Laboratory Animal Care. All animal studies were approved by the Animal Research Committee of the William S. Middleton Memorial Veterans Hospital.

A neutropenic, corticosteroid immunosuppressed mouse IPA model was utilized, as previously described (19, 20). Briefly, mice were rendered neutropenic (polymorph nuclear cell counts, <100/mm^3^) by the subcutaneous (s.c.) injection of 150 mg/kg cyclophosphamide on Days −4 and −1 and +3 to ensure the maintenance of neutropenia until the end of study (96 h). Additionally, cortisone acetate was administered at 250 mg/kg s.c. on Day −1. Throughout the 4-day experiment, mice were also given ceftazidime at 50 mg/kg/day s.c. to prevent opportunistic bacterial infection.

Organisms were prepared by sub-culturing on potato dextrose agar 5 days prior to infection and incubated at 37°C. On the day of infection, the inoculum was prepared by flooding the culture plate with 5 mL of normal saline containing 0.05% Tween 20. Gentle agitation was used to release the conidia. The conidial suspension was collected and quantified by hemocytometer (Bright-Line, Hausser Scientific, Horsham, PA, USA). The suspension was diluted in saline to a final concentration of 1 × 10^7^ conidia/mL. Viability was confirmed by plating the suspension and performing CFU counts. Infection was produced in animals using an aspiration pneumonia model that has been successfully utilized in previous studies (17, 19). Briefly, mice were anesthetized with a combination of ketamine and xylazine. Fifty microliters of the conidia suspension was pipetted into the anterior nares with mice held upright to allow aspiration into the lungs. Drug treatment commenced 2 h after the initiation of infection, and the treatment period was 96 h.

Aspergillus fungal burden was determined by real-time quantitative PCR (qPCR) using previously reported methods (17, 18). Briefly, animals were euthanized if showing signs of distress or at the end of the 96-h experiment by CO_2_ asphyxiation. Both lungs were immediately removed by aseptic technique and frozen at −80°C in 2.0-mL sterile polypropylene screw top tubes and then lyophilized overnight using a VirTis Freezemobile 25ES (SP Industries VirTis. Warminster, PA). Samples were homogenized with 2.3-mm Zirconia/silica beads (Research Products International, Mt. Prospect, IL) via bead beating for one minute at 4,350 rpm using a BeadBug six homogenizer (Benchmark Scientific, Sayreville, NJ). DNA was extracted using the E.Z.N.A. Plant and Fungal DNA kit (Omega Bio-Tek, Norcross, GA) with the following alterations to standard kit protocol: The incubation period after addition of buffer FG1 was increased from 10 to 30 min and after addition of buffer FG2, only 400-μL of sample was used downstream to avoid clogging of the kit columns. At the end of the kit protocol, DNA was extracted in 100-μL of kit elution buffer and used for real-time PCR analysis.

Samples were assayed in triplicate using a Bio-Rad CFX96 real-time system (Bio-Rad, Hercules, CA, USA). A single-copy gene, FKS1, was chosen for quantitation. IDT PrimeTime Std qPCR Assay was used with a primer probe ratio of 2:1. The following sequences were in the standard assay: forward primer (5′-GCCTGGTAGTGAAGCTGAGCGT-3′), reverse primer (5′-CGGTGAATGTAGGCATGTTGTCC-3′), and probe (5′−56-FAM- TCACTCTCT/ZEN/ACCCCCATGCCCGAGCC-3IABkFQ-3′) (Integrated DNA Technologies, Coralville, IA, USA). PrimeTime Gene Expression Master Mix was used for qPCR (Integrated DNA Technologies, Coralville, IA, USA) using 20 µL total reaction volume, with 5 μL of DNA sample used per well. PCR protocol was as follows: 1 cycle of 95°C for 3 min, then 45 cycles of two-step PCR, 95°C for 5 s, and then 60°C for 1 min. Standard curves were generated using dilutions of known concentrations of plasmid pFKS1_300BP containing the target region of the above primer-probe set. Samples were analyzed using the BioRad CFX Maestro Software Version 2.3 (Bio-Rad Life Sciences, Hercules, CA).

Posaconazole pharmacokinetics were completed in infected mice to assure concentrations estimate profiles observed during subsequent treatment studies. Animal and organism preparation are described above. Prior studies with this antifungal in this model were used to guide dose levels. Doses utilized included 2.5, 10, 40, and 160 mg/kg. Drug was administered by oral gavage. Plasma was collected from infected mice using three per dose/time point and seven time points (1, 4, 8, 12, 24, 30, and 36 h) to describe the pharmacokinetics via LCMS. Protein binding was assessed to allow comparison of mouse and human free-drug concentrations at concentrations of 1, 5, and 25 mg/L.

### LCMS assay

Drug assay was developed and validated using liquid chromatography mass spectrometry (LCMS/MS). Assay limit of detection and variability reported in Table S1.

### Protein binding assay

We assessed binding at three concentrations. A protein-bound drug was assessed following incubation in mouse serum. All samples were passed through the ultrafiltration columns, and eluate concentrations were measured by LCMS assay. The bound fraction of drug was calculated:


$$\%\;bound\;=\;\frac{\left[serum\right]-\left[ultrafiltrate\right]}{\left[serum\right]}\times 100$$

We utilized both free and total drug pharmacokinetic assay results in PK/PD analyses. A time-ordered data set was constructed using the pooled PK data collected. A non-compartmental PK model was fit to the data set.

We assessed burden after 96 h of posaconazole therapy using quantitative organ homogenate PCR. This study duration was based upon the amount of time needed for sufficient control growth in the model. A sigmoid Emax model was used to identify the magnitude of dose and AUC/MIC associated with a net stasis endpoint (suppressing fungal growth compared with the fungal burden at the start of therapy) and the log_10_ unit CFU reduction in burden (compared to start of therapy) for posaconazole and each organism. The treatment endpoint associated with the humanized posaconazole regimen was defined as a dose of 7 mg/kg/day based upon the AUC of 37.9 mg*h/L (42% CV) for the human AUC for the posaconazole DRT 300 mg tablet (AUC_0-24h_ Food and Drug Administration [FDA] label table 13) (11).

### Treatment regimens

Humanized regimens were defined with mouse plasma PK as described above.

### Outcome assessment and determination of PK/PD endpoint

Quantitative PCR of lung homogenates provides an accurate assessment of viable fungi and animal survival. The limit of detection is approximately 100 conidial equivalents, and the nucleic acid tissue extraction efficiency is more than 90%. A sigmoid Emax model was used to identify the magnitude of the dose and fAUC/MIC associated with a stasis endpoint and the log_10_ CFU unit reduction in fungal burden (compared with start of therapy). The organism burden in the model using humanized posaconazole against susceptible isolates was used to define the endpoint associated with clinical success. Treatment against resistant strains is expected to be less effective. These data points will serve as negative controls.

Using the pharmacokinetic parameters obtained from the pharmacokinetic model, total drug plasma concentration-time profiles were generated for each dosing regimen studied. Area under the concentration-time curve over 24 h (AUC_0-24h_) was determined using numeric integration. Using the challenge isolates MIC value, AUC_0-24h_ relative to MIC was determined for each dosing regimen. The relationships between change in CFU from baseline and AUC/MIC were evaluated. Hill-type models were then fit to the data using nonlinear least-squares regression. Both visual inspection of the data and the coefficient of determination (R^2^) were used to assess model fit.

### Identification of population PK models

Literature searches were conducted using PubMed to identify PK data to support assessing the PK/PD target attainment for posaconazole. Search terms included “posaconazole,” combined with terms, such as “pharmacokinetics,” “population pharmacokinetics,” “pharmacokinetic model,” and “population pharmacokinetic model.” Search results also had their bibliographies reviewed to determine if any additional publications could be identified. The United States FDA approval package for posaconazole was evaluated for pertinent information. Results that provided posaconazole PK information but did not develop a population PK model were considered for qualification of the chosen models. Non-compartmental analyses and physiologically-based population PK models were not considered. Results were limited to those in adult humans and publications in English. Preference was given to publications describing the development of population PK models using PK data from target patient populations of interest, which included those with pulmonary fungal infections.

Criteria that were used to discriminate among candidate models are the following:

- Evaluation of individual and population mean parameter estimates and their precision (e.g., standard error of the mean [SEM]);
- Graphical examination of standard goodness-of-fit diagnostic plots and the observed versus individual predicted drug concentration-time profiles, as a measure of model precision;
- Relevant covariates assessed (renal function for clearance; body size for volume);
- A structural and statistical model that is reproducible from the original publication; and
- Data based on a patient population that is generalizable to patients with target infections of interest.

If PK data from the target patient populations were not found and PK data were instead obtained from healthy subjects, inflation of the variance in mean population PK model parameter estimates was considered to more closely approximate PK from these target patient populations.

Candidate population PK models were then selected by evaluating their ability to recapture relevant data from the literature (i.e., the data used to build the model as well as data from other studies, regardless of the methods used to analyze that data). The selection/qualification process included the following steps, which were conducted separately for each study of interest:

1. Conversion of the model to a format suitable for the conduct of model-based simulations;
2. Creation of a hypothetical population of subjects with characteristics consistent with those reported for the study of interest (e.g., body weight, renal function, age, etc.);
3. Simulation of concentration-time profiles for the hypothetical subject population; and,
4. Comparison of the simulated concentration-time curves to the data reported in the study of interest. Note that this could also include comparison of PK parameters derived from the concentration-time curve such as area under the concentration-time curve or maximum plasma concentration.

Given the likely variability to be observed in the studies, it was not expected that a single model could robustly capture all of the PK data found in the literature for a given agent. Thus, a holistic approach was taken in order to select population PK models that best captured the broadest range of applicable PK data. Given the details regarding the subject-level data were not always available, it was necessary to rely primarily on model-based simulations to identify or define population PK models that achieved this goal.

All model qualification and simulation activities were conducted using R Version 4.0.4 (3).

### Generation of simulated patients

Using the population PK models selected for posaconazole and R version 4.04, populations of 5,000 simulated patients were generated using random selection of patient covariates predictive of posaconazole PK using distributions of these variables reported for the target patient population.

Typical PK parameter values for each simulated patient were calculated using demographic values in conjunction with the fixed effect parameter estimates for the population PK models for posaconazole. Individual PK parameter values for each simulated patient were then generated by applying an individual-specific random effect (η) to each simulated patient’s typical PK value. Each simulated patient’s η value was drawn from a log-normal distribution with a mean of zero and a variance based on the reported interindividual variability in the PK parameters of interest from the selected population PK models. Thus, for any cohort of simulated patients with the same demographics, the individually generated PK parameters resulted in distinct simulated patients.

### Generation of drug exposures in simulated patients

Using the population PK model and above-described individual PK parameter values for posaconazole for each simulated patient, free drug plasma concentration-time profiles from 0 to 264 h after administration of the posaconazole 300 mg PO q12h on Day 1 followed by 300 mg PO q24h on Day 2 and thereafter were generated. Free drug plasma concentrations were determined using plasma protein binding estimates of 98.7% (62). The 24-h free drug plasma AUC values were calculated by integration of the plasma-concentration time curves and the patterns over Days 1 to 11 were summarized. To assess PK/PD target attainment relative to 96-h free drug plasma AUC/MIC targets, 96-h AUC values on Days 1 to 4 (i.e., 0 to 96 h) and Days 8 to 11 (i.e., 168 to 264 h) among simulated patients were calculated.

For each simulated patient and combination of dosing regimen and protein binding estimate, the 96-h free drug plasma AUC/MIC for a fixed MIC value was determined by dividing the free-drug plasma AUC value by the MIC value. This calculation was performed over the ranges of posaconazole MIC distributions for the collection of *A. fumigatus* isolates described above.

### PK/PD target attainment

Percent probabilities of PK/PD target attainment by posaconazole MIC and overall on Days 1 to 4 and Days 8 to 11 were carried out based on the average 96-h free drug plasma AUC values for each agent and the assessment of median and randomly assigned free-drug plasma AUC/MIC targets for *A. fumigatus* among simulated patients after administration of the dosing regimens described above. Median and randomly assigned free drug plasma AUC/MIC targets associated with net fungal stasis were evaluated. For posaconazole, percent probabilities of PK/PD target attainment were assessed among simulated patients in both fasted and fed states, evenly split into simulated patients in fasted and fed states. For the assessment based on randomly assigned AUC/MIC targets, posaconazole free drug plasma AUC/MIC targets were randomly assigned to simulated patients based on an estimated log-normal distribution of free-drug plasma AUC/MIC targets associated with net fungal stasis. The observed mean and standard deviation on a natural log scale among the free-drug plasma AUC/MIC targets were used as the parameters for the log-normal distribution of targets. The distributions of free drug plasma AUC/MIC targets were truncated on the log scale such that randomly selected targets are all within two standard deviations of the mean values.

Of these approaches, random assignment of free drug plasma AUC/MIC targets was preferred, and the results of PK/PD target attainment analyses based on this approach were emphasized. The basis for this preference was twofold. First, its incorporation of variability of targets across simulated patients that cannot be carried out with fixed target values and second, its unbiased nature relative to yet another alternative, which is assignment of a high percentile as a target value.

With respect to the PK/PD target attainment analyses, candidate susceptible breakpoints for posaconazole against *A. fumigatus* were based upon the highest MIC value at which the percent probabilities of PK/PD target attainment were ≥90% based on median and randomly assigned 96-h free drug plasma AUC/MIC targets associated with net fungal stasis.

## SUPPLEMENTAL MATERIAL

Supplemental Material aac.01643-25-s0001.pdf Tables S1 to S6; Fig. S1 to S8.

ASM does not own the copyrights to Supplemental Material that may be linked to, or accessed through, an article. The authors have granted ASM a non-exclusive, world-wide license to publish the Supplemental Material files. Please contact the corresponding author directly for reuse.
