# Supplementary material for: Evaluation of an in vivo pulmonary aspergillosis model for triazole susceptibility breakpoint development
Source: Antimicrob Agents Chemother. 2026 Jan 30;70(3):e01643-25. doi: 10.1128/aac.01643-25 (PMC12959153; doi:10.1128/aac.01643-25)
Supplement: Supplemental Material — Tables S1 to S6; Fig. S1 to S8. [file aac.01643-25-s0001.pdf]

## Supplemental Information

## Supplemental Table S1. LCMS Precision, Accuracy, Calibration

**Plasma (High): Inter-Run Posaconazole Precision and Accuracy**

| Quality Control | | LQ1 | 1 | 2 | 3 |
|---|---|---|---|---|---|
| Conc. (mg/L) | | 0.0500 | 0.1750 | 1.5000 | 15.0000 |
| 1/26/2024 | 1a | 0.049 | 0.169 | 1.470 | 15.10 |
| 1/26/2024 | 1c | 0.052 | 0.166 | 1.490 | 14.80 |
| 1/26/2024 | 2a | 0.054 | 0.168 | 1.510 | 14.70 |
| 1/26/2024 | 2f | 0.056 | 0.164 | 1.490 | 14.70 |
| Mean | | 0.053 | 0.167 | 1.490 | 14.83 |
| Std Dev | | 0.003 | 0.002 | 0.016 | 0.19 |
| %CV | | 5.13% | 1.33% | 1.10% | 1.28% |
| %A | | 105.5% | 95.3% | 99.3% | 98.8% |

**Plasma (High): Posaconazole Calibration Curve**

| Method Dynamic Range | |
|---|---|
| LLOQ (mg/L) | 0.050 |
| Max (mg/L) | 25.000 |

| Operator | Date | Run | Regression Model | Slope | Intercept | Correlation Coefficient |
|---|---|---|---|---|---|---|
| CVB | 1/26/2024 | 1 | Linear $1/x^2$ | 0.433 | -8.54E-04 | 0.9997 |
| CVB | 1/26/2024 | 2 | Linear $1/x^2$ | 0.436 | -1.45E-03 | 0.9994 |
| | | | | | Min | 0.9994 |
| | | | | | Max | 0.9997 |

**Plasma (Low): Inter-Run Posaconazole Precision and Accuracy**

| Quality Control | | LQ1 | 1 | 2 | 3 |
|---|---|---|---|---|---|
| Conc. (mg/L) | | 0.0025 | 0.00750 | 0.0350 | 0.1250 |
| 1/30/2024 | 6a | 0.00269 | 0.0071 | 0.0349 | 0.132 |
| 1/30/2024 | 6f | 0.00287 | 0.0073 | 0.0335 | 0.128 |
| 1/31/2024 | 7a | 0.00261 | 0.0069 | 0.0356 | 0.126 |
| 1/31/2024 | 7b | 0.00261 | 0.0077 | 0.0340 | 0.128 |

**Plasma (Low): Posaconazole Calibration Curve**

| Method Dynamic Range | |
|---|---|
| LLOQ (mg/L) | 0.0025 |
| Max (mg/L) | 0.2000 |

| Operator | Date | Run | Regression Model | Slope | Intercept | Correlation Coefficient |
|---|---|---|---|---|---|---|

| | | | | | | | | | | | |
|---|---|---|---|---|---|---|---|---|---|---|---|
| Mean | 0.00270 | 0.00722 | 0.0345 | 0.129 | CVB | 1/30/2024 | 6 | Linear $1/x^2$ | 9.750 | 1.47E-03 | 0.9983 |
| Std Dev | 0.00012 | 0.00032 | 0.0009 | 0.003 | CVB | 1/31/2024 | 7 | Linear $1/x^2$ | 9.750 | 9.42E-04 | 0.9987 |
| %CV | 4.55% | 4.47% | 2.71% | 1.96% | | | | | | | |
| %A | 107.8% | 96.3% | 98.6% | 102.8% | | | | | | Min | 0.9983 |
| | | | | | | | | | | Max | 0.9987 |

## Table S2. Posaconazole LCMS assay QC and protein binding data

| Starting Posaconazole Concentration | | Observed Posaconazole Concentration (µg/mL) | | % Posaconazole Bound | | | % Recovery | | |
|---|---|---|---|---|---|---|---|---|---|
| | | Total | Free | Trial | Average | Std. Dev | Trial | Average | Std. Dev |
| 1.00 µg/mL | A | 0.9585 | 0.0040 | 99.6% | 99.5% | 0.1% | 96.6% | 96.87% | 0.61% |
| | B | 0.9520 | 0.0052 | 99.5% | | | 96.4% | | |
| | C | N/A | N/A | N/A | | | N/A | | |
| | D | 0.9665 | 0.0044 | 99.5% | | | 97.6% | | |
| 5.00 µg/mL | A | 4.975 | 0.038 | 99.2% | 99.4% | 0.1% | 96.6% | 97.5% | 2.0% |
| | B | 5.020 | 0.025 | 99.5% | | | 96.5% | | |
| | C | 5.015 | 0.026 | 99.5% | | | 96.5% | | |
| | D | 5.225 | 0.028 | 99.5% | | | 100.6% | | |
| 25.0 µg/mL | A | 24.000 | 0.149 | 99.4% | 99.4% | 0.1% | 95.9% | 95.9% | 1.1% |
| | B | 24.250 | 0.136 | 99.4% | | | 96.7% | | |
| | C | 24.300 | 0.122 | 99.5% | | | 96.7% | | |
| | D | 23.550 | 0.158 | 99.3% | | | 94.3% | | |

## Supplemental Table S3 1 Log Kill Endpoint Exposures

| | | 1-log kill | | | |
|---|---|---|---|---|---|
| Strain | MIC | 1-log kill dose (mg/kg/24h) | 96h Total AUC (mg*h/L) | 96h Total AUC/MIC | 96h fAUC/MIC |
| 1070650 | 0.25 | 4.29 | 99.87 | 399.50 | 2.40 |
| 1072954 | 0.25 | 6.31 | 145.54 | 582.15 | 3.49 |
| 1053216 | 0.5 | 6.17 | 142.25 | 284.49 | 1.71 |
| 1072941 | 0.5 | 3.94 | 91.87 | 183.75 | 1.10 |
| 1077395 | 1 | | | | |
| 1100802 | 1 | 20.13 | 408.81 | 408.81 | 2.45 |
| F16216 | 1 | | | | |
| F13747 | 1 | 11.38 | 253.25 | 253.25 | 1.52 |
| Mean | | 8.70 | 190.26 | 351.99 | 2.11 |
| Median | | 6.24 | 143.89 | 341.99 | 2.05 |
| Stdev | | 6.20 | 121.56 | 142.18 | 0.85 |
| | | | | | |
| Standard Error | | 2.19 | 42.98 | 50.27 | 0.30 |
| Zc | | 1.96 | 1.96 | 1.96 | 1.96 |
| margin of error | | 4.30 | 84.24 | 98.52 | 0.59 |
| 95% CI Lower bound | | 4.41 | 106.03 | 253.47 | 1.52 |
| 95% CI Upper bound | | 13.00 | 274.50 | 450.51 | 2.70 |

## Table S4. *Aspergillus* Phenotypes and CYP genotype

| Isolate | Site Code | State and/or Country | MIC according to CLSI method (µg/mL): | | | | *CYP* mutations[a]: | |
|---|---|---|---|---|---|---|---|---|
| | | | Isavuconazole | Posaconazole | Voriconazole | Itraconazole | *cyp*51A | *cyp*51B |
| 782671 | 127 | Ireland | 8 | 1 | 2 | 4 | L98H,TR34 | Wild type |
| 801380 | 090 | France | 8 | 1 | 2 | 4 | L98H,TR34 | Wild type |
| 867022 | 150 | Germany | >8 | 1 | >8 | 1 | Y121F,T289A | Wild type |
| 884225 | 024 | TX, USA | 2 | 0.5 | 1 | 1 | Wild type | Wild type |
| 913224 | 025 | TX, USA | 2 | 0.12 | 1 | 0.5 | I242V | Wild type |
| 913683 | 377 | Italy | 1 | 1 | 0.5 | >8 | F219I | Wild type |
| 920125 | 147 | Canada | 2 | 0.5 | 1 | 0.5 | Wild type | Wild type |
| 931697 | 603 | Thailand | 1 | 1 | 1 | 8 | Wild type | Wild type |
| 941455 | 107 | KY, USA | 2 | 0.5 | 0.5 | 2 | Wild type | Wild type |
| 973761 | 377 | Italy | 4 | 1 | 2 | 8 | L98H,TR34 | Wild type |
| 1023697 | 614 | Australia | 2 | 0.25 | 0.5 | 0.5 | Wild type | Wild type |
| 1023703 | 614 | Australia | 2 | 0.5 | 0.5 | 0.5 | Wild type | Wild type |
| 1024715 | 025 | TX, USA | 2 | 0.5 | 0.5 | 0.5 | Wild type | Wild type |
| 1027852 | 136 | NY, USA | 2 | 0.5 | 0.5 | 1 | Wild type | Wild type |
| 1027857 | 017 | NY, USA | 2 | 0.5 | 0.5 | 1 | Wild type | Wild type |
| 1027858 | 017 | NY, USA | 2 | 0.5 | 1 | 1 | Wild type | Q42L |
| 1027892 | 377 | Italy | 2 | 0.5 | 0.5 | 1 | Wild type | Wild type |
| 1027894 | 377 | Italy | 2 | 0.5 | 0.5 | 1 | Wild type | Wild type |

| Isolate | Site Code | State and/or Country | MIC according to CLSI method (µg/mL): | | | | *CYP* mutations[a]: | |
|---|---|---|---|---|---|---|---|---|
| | | | Isavuconazole | Posaconazole | Voriconazole | Itraconazole | *cyp*51A | *cyp*51B |
| 1027899 | 377 | Italy | 2 | 0.25 | 0.5 | 0.5 | Wild type | Wild type |
| 1027901 | 377 | Italy | 4 | 1 | 2 | 4 | L98H,TR34 | Wild type |
| 1027905 | 377 | Italy | 8 | 1 | 2 | 8 | L98H,TR34 | Wild type |
| 1027910 | 377 | Italy | 8 | 1 | 4 | 4 | L98H,TR34 | Wild type |
| 1032153 | 603 | Thailand | 2 | 0.25 | 0.5 | 1 | F46Y,M172V,N248T,D255E,E427K | Q42L |
| 1046494 | 150 | Germany | 2 | 0.5 | 0.5 | 1 | Wild type | Wild type |
| 1046949 | 146 | CA, USA | 2 | 0.25 | 0.5 | 1 | Wild type | Wild type |
| 1047452 | 002 | IN, USA | 2 | 0.5 | 0.5 | 0.5 | Wild type | Wild type |
| 1050245 | 129 | NJ, USA | 2 | 0.25 | 0.5 | 1 | Wild type | Wild type |
| 1051037 | 002 | IN, USA | 1 | 1 | 1 | 2 | I242V | Wild type |
| 1051070 | 150 | Germany | 2 | 0.5 | 0.5 | 1 | Wild type | Wild type |
| 1051331 | 129 | NJ, USA | 4 | 0.5 | 2 | 2 | Wild type | Q42L |
| 1051334 | 129 | NJ, USA | 2 | 0.5 | 0.5 | 1 | Wild type | Q42L,D71A |
| 1053208 | 032 | Canada | 2 | 0.5 | 0.5 | 0.5 | Wild type | Wild type |
| 1053211 | 032 | Canada | 2 | 0.5 | 0.5 | 1 | Wild type | Wild type |
| 1053216 | 032 | Canada | 1 | 0.5 | 0.5 | 2 | I242V | Wild type |
| 1065541 | 203 | Australia | 1 | 0.25 | 2 | 1 | Wild type | Q42L |
| 1065544 | 203 | Australia | 2 | 0.5 | 0.5 | 1 | Wild type | Wild type |
| 1070622 | 122 | VT, USA | 2 | 0.5 | 1 | 2 | F46Y,M172V,E427K | Wild type |

| Isolate | Site Code | State and/or Country | MIC according to CLSI method (μg/mL): | | | | *CYP* mutations[a]: | |
|---|---|---|---|---|---|---|---|---|
| | | | Isavuconazole | Posaconazole | Voriconazole | Itraconazole | *cyp*51A | *cyp*51B |
| 1070650 | 122 | VT, USA | 2 | 0.25 | 0.5 | 1 | A9T | Wild type |
| 1070868 | 129 | NJ, USA | 2 | 0.5 | 0.5 | 1 | Wild type | K147N,K148Q |
| 1072941 | 302 | Czech Republic | 0.5 | 0.25 | 0.5 | 2 | Wild type | Wild type |
| 1072943 | 302 | Czech Republic | 2 | 0.5 | 1 | 2 | F46Y,M172V,N248T,D255E,E427K | Wild type |
| 1072944 | 302 | Czech Republic | 0.5 | 0.25 | 0.5 | 2 | Wild type | Wild type |
| 1072952 | 302 | Czech Republic | 2 | 0.25 | 0.5 | 0.5 | Wild type | Wild type |
| 1072954 | 302 | Czech Republic | 0.5 | 0.25 | 1 | 2 | Wild type | Wild type |
| 1072958 | 302 | Czech Republic | 2 | 0.25 | 0.5 | 1 | Wild type | Wild type |
| 1073599 | 030 | VA, USA | 1 | 0.5 | 0.5 | 2 | A9T | Wild type |
| 1075598 | 131 | Belgium | 4 | 1 | 2 | 4 | L98H,TR34 | Wild type |
| 1077391 | 377 | Italy | 8 | 1 | 2 | 8 | L98H,TR34 | Wild type |
| 1077392 | 377 | Italy | >8 | 4 | >8 | >8 | L98H,TR34 | Wild type |
| 1077393 | 377 | Italy | 4 | 1 | 2 | 4 | L98H,TR34 | Wild type |
| 1077394 | 377 | Italy | 4 | 0.5 | 1 | 4 | L98H,TR34 | Wild type |

| Isolate | Site Code | State and/or Country | MIC according to CLSI method (μg/mL): | | | | *CYP* mutations[a]: | |
|---|---|---|---|---|---|---|---|---|
| | | | Isavuconazole | Posaconazole | Voriconazole | Itraconazole | *cyp*51A | *cyp*51B |
| 1077395 | 377 | Italy | 4 | 1 | 2 | 4 | L98H,TR34 | Wild type |
| 1085981 | 003 | MI, USA | 2 | 0.25 | 0.5 | 0.5 | Wild type | Wild type |
| 1087624 | 806 | VA, USA | 1 | 0.5 | 0.5 | 2 | I242V | Wild type |
| 1100802 | 329 | Slovenia | 4 | 0.5 | 2 | >8 | L98H,TR34 | Wild type |
| 1102402 | 129 | NJ, USA | 2 | 0.5 | 1 | 2 | Wild type | Q42L |
| 1116483 | 603 | Thailand | 1 | 0.5 | 0.5 | 2 | Wild type | Wild type |
| 1118418 | 614 | Australia | 0.5 | 0.5 | 0.5 | 2 | Wild type | Wild type |
| 1121859 | 303 | UK | 4 | 0.5 | 2 | >8 | L98H,TR34 | Wild type |
| 1122732 | 377 | Italy | 4 | 0.5 | 2 | 2 | L98H,TR34 | Wild type |
| 1122735 | 377 | Italy | 2 | 0.5 | 2 | 2 | L98H,TR34 | Wild type |
| 1124368 | 136 | NY, USA | 1 | 1 | 0.5 | 2 | Wild type | Q42L |
| 1131576 | 806 | VA, USA | >8 | 0.5 | 4 | >8 | G448S | Wild type |
| 1131591 | 131 | Belgium | >8 | 0.5 | >8 | 8 | Y121F,M172I,T289A,G448S,TR46 | Wild type |
| 1151434 | 002 | IN, USA | 4 | 1 | 2 | 4 | Wild type | Wild type |
| 1164541 | 017 | NY, USA | 1 | 0.5 | 0.5 | 2 | Wild type | Wild type |
| 1169542 | 129 | NJ, USA | 2 | 0.5 | 1 | 2 | Wild type | Q42L |
| 1169545 | 129 | NJ, USA | 0.5 | 0.25 | 0.5 | 2 | Wild type | Q42L |
| 1170537 | 052 | MA, USA | 0.5 | 0.5 | 0.5 | 2 | N248K | Wild type |
| 1170842 | 614 | Australia | 2 | 0.5 | 1 | 2 | Wild type | K82Q,F149V,P383L |

| Isolate | Site Code | State and/or Country | MIC according to CLSI method (µg/mL): | | | | *CYP* mutations[a]: | |
|---|---|---|---|---|---|---|---|---|
| | | | Isavuconazole | Posaconazole | Voriconazole | Itraconazole | *cyp*51A | *cyp*51B |
| 1173392 | 122 | VT, USA | 1 | 0.5 | 0.5 | 2 | F46Y,M172V,N248T,D255E,E427K | Wild type |
| 1173409 | 122 | VT, USA | 1 | 0.5 | 0.5 | 2 | Wild type | Wild type |
| 1174066 | 091 | France | 1 | 0.5 | 0.5 | 2 | Wild type | Wild type |
| 1175751 | 260 | New Zealand | >8 | 8 | 8 | >8 | G138C | Wild type |
| 1175944 | 129 | NJ, USA | 1 | 1 | 0.5 | 2 | Wild type | Wild type |
| 1177980 | 068 | Turkey | 1 | 0.5 | 1 | 2 | Wild type | Wild type |
| 1177982 | 068 | Turkey | 2 | 1 | 2 | 2 | Wild type | Wild type |
| 1179124 | 303 | UK | 4 | 1 | 2 | 4 | L98H,TR34 | Wild type |
| 1188596 | 456 | AL, USA | 1 | 0.25 | 0.5 | 2 | I242V | Wild type |
| 1192458 | 129 | NJ, USA | 2 | 0.5 | 2 | 4 | Wild type | Q42L |
| 1195605 | 425 | KS, USA | 2 | 0.5 | 1 | 2 | Wild type | Wild type |
| 1197089 | 329 | Slovenia | 1 | 0.25 | 0.5 | 2 | Wild type | Wild type |
| 1197175 | 052 | MA, USA | 1 | 1 | 1 | 2 | Wild type | Wild type |
| 1200487 | 129 | NJ, USA | 2 | 0.5 | 1 | 2 | Wild type | Wild type |
| 1201295 | 091 | France | 0.5 | 0.5 | 0.5 | 2 | K67Q | Wild type |
| 1201307 | 091 | France | >8 | 2 | >8 | >8 | H147Y | Wild type |
| 1201314 | 091 | France | 1 | 0.5 | 0.5 | 2 | Wild type | Wild type |
| 1201318 | 091 | France | 1 | 0.5 | 0.5 | 2 | Wild type | F149V |
| 1201870 | 003 | MI, USA | 2 | 1 | 2 | 2 | K67Q | Wild type |

| Isolate | Site Code | State and/or Country | MIC according to CLSI method (µg/mL): | | | | *CYP* mutations[a]: | |
|---|---|---|---|---|---|---|---|---|
| | | | Isavuconazole | Posaconazole | Voriconazole | Itraconazole | *cyp*51A | *cyp*51B |
| 1205394 | 017 | NY, USA | 1 | 0.5 | 0.5 | 2 | Wild type | Wild type |
| 1209363 | 002 | IN, USA | 0.5 | 0.25 | 0.5 | 2 | Wild type | Wild type |
| 1213020 | 302 | Czech Republic | 1 | 0.5 | 0.5 | 2 | F46Y,D172V,E427K | Wild type |
| 1213028 | 302 | Czech Republic | 4 | 1 | 2 | >8 | L98H,TR34 | Wild type |
| 1213031 | 302 | Czech Republic | 1 | 0.5 | 0.5 | 2 | F46Y,D172V,E427K | Wild type |
| 1213032 | 302 | Czech Republic | 1 | 0.5 | 0.5 | 2 | D172V | Wild type |
| 1213033 | 302 | Czech Republic | 0.5 | 0.5 | 1 | 2 | Wild type | Wild type |
| 1214571 | 806 | VA, USA | 1 | 0.5 | 0.5 | 2 | I242V | Wild type |
| 1214586 | 806 | VA, USA | 1 | 0.5 | 0.5 | 2 | Wild type | Wild type |
| 1218415 | 002 | IN, USA | 1 | 0.5 | 1 | 4 | I242V | Q42L,S501Q |
| 1218426 | 002 | IN, USA | 2 | 0.5 | 1 | 1 | I242V | Q42L,S501Q |
| 1218553 | 107 | KY, USA | 1 | 0.5 | 1 | 2 | Wild type | Wild type |
| 1218562 | 107 | KY, USA | 1 | 0.5 | 1 | 2 | Wild type | Wild type |
| 1218797 | 377 | Italy | 1 | 0.25 | 0.5 | 2 | Wild type | Wild type |
| 1246708 | 131 | Belgium | 8 | 1 | 4 | >8 | L98H,TR34 | Wild type |

| Isolate | Site Code | State and/or Country | MIC according to CLSI method (µg/mL): | | | | *CYP* mutations[a]: | |
|---|---|---|---|---|---|---|---|---|
| | | | Isavuconazole | Posaconazole | Voriconazole | Itraconazole | *cyp*51A | *cyp*51B |
| 1246715 | 131 | Belgium | >8 | 1 | 8 | >8 | L98H,TR34 | Wild type |
| 1251689 | 091 | France | 8 | 1 | 4 | >8 | L98H,TR34 | Wild type |
| 1258509 | 806 | VA, USA | >8 | 1 | >8 | >8 | G448S | Q42L |
| 1258945 | 336 | Hungary | 0.5 | 0.12 | 0.5 | 2 | Wild type | Wild type |
| 1262011 | 260 | New Zealand | >8 | 1 | >8 | >8 | Y121F,T289A | Wild type |

Table S5 shows the posaconazole publications identified for model qualification. As shown in Figure S1, the population PK model from Iwasa *et al*. was found to robustly capture the data from Days 1 and 8 from the study by Duarte *et al*. [2], a Phase 1b study in patients with myelodysplastic syndromes or acute myelogenous leukemia (MDS/AML) receiving posaconazole tablets as prophylaxis.

When qualified with data from a study by Petitcollin *et al*. [3] in patients with hematological malignancies receiving prophylaxis with posaconazole tablets, the Iwasa model was found to capture the data well (Figure S2). There was a tendency for the observed concentrations to be below the median simulated concentration profile, which may reflect our inability to account for the impact of treatment discontinuation, which was not described in the publication by Petitcollin *et al*.

As shown in Figure S3, the Iwasa model was qualified with data from Cornely *et al*. [4], a Phase 3 study in patients at high risk of invasive fungal infections who received IV posaconazole prophylaxis. Overall, the Iwasa model was found to overpredict the observed mean concentrations. However, the values were predominantly contained within the 90% prediction intervals.

The Iwasa model was found to overpredict the observed concentrations from Sime *et al*. [5], a Phase 1 study evaluating single doses of IV posaconazole in intensive care unit (ICU) patients (Figure S4). This overprediction was expected given that Sime *et al*. ICU patients have altered PK compared to that reported elsewhere for non-ICU patients. Similar trends were seen when evaluating the distribution of predicted CL and volume of distribution at steady state (Vss) (Figure S4).

When qualified with data from Liu *et al*. [6], a study in patients with hematological malignancies receiving posaconazole tablets as prophylaxis, the Iwasa model captured the data from Days 1 and 8 robustly (Figure S5).

As shown in Figure S6, the Iwasa model was found to adequately capture the concentration-time data from Wasmann *et al*. [7], a Phase 1 study evaluating single IV doses of posaconazole in normal weight and obese subjects. Simulated CL values by weight generated using the Iwasa model matched up well with the values reported by Wasmann *et al*. (Figure S6).

The Iwasa model captured the data from Wu *et al*. [8], a Phase 1b/3 study in Asian patients at high risk of invasive fungal infections receiving IV posaconazole for prophylaxis, on Day 10 robustly, as shown in Figure S7. The Iwasa model was also found to capture the impact of age on posaconazole CL reliably using data from Dvorackova *et al*. [9], a study in adult lung transplant patients receiving posaconazole tablets as prophylaxis or treatment (Figure S8).

**Table S5. Posaconazole population PK models from the literature**

| Citation | Patient population[a] | Model (covariates) |
|---|---|---|
| Dvorackova *et al*. Antibiotics *(Basel)*. 2023;12(9):1399. [9] | Lung transplant patients (n=32) | Linear, 1-compartment, first-order absorption (age) |
| Iwasa *et al*. J Clin Pharmacol 2023;63(4):421-434. [1] | Healthy adults and patients with deep-seated fungal infections (n=522) | Linear, 2-compartment, sequential zero-order and first-order absorption (disease state, body weight, formulation, food status, dosing regimen [single vs. multiple dose studies]) |
| Jansen *et al*. Clin Microbiol Infect 2022;28:1003-1009. [10] | Patients with a hematological malignancy or those receiving induction chemotherapy for AML/MDS (n=23) | Linear, 2-compartment, sequential zero-order and first-order absorption (body weight, severe mucositis) |
| Pena-Lorenzo *et al*. Eur J Pharm Sci 2022;168:106049. [11] | Stem cell transplant patients (n=36) | Linear- 1-compartment, sequential zero-order and first-order absorption (sex and total protein in serum) |

**Table S5. Posaconazole population PK models from the literature**

| Citation | Patient population[a] | Model (covariates) |
|---|---|---|
| Petitcollin *et al*. Antimicrob Agents Chemother. 2017;61(11):e01166-17. [3] | Hospitalized patients with hematological malignancies (n=49) | Linear, 1-compartment model (none) |
| van Iersel *et al*. Antimicrob Agents Chemother 2018;62:e02465-17. [12] | Healthy adults and those receiving posaconazole for prophylaxis (n=335) | Linear, 1-compartment, sequential zero-order and first-order absorption (disease state, body weight, formulation, food status, dosing regimen [single vs. multiple dose studies]) |
| Wasmann *et al*. J Antimicrob Chemother 2020; 75:1006-1013. [7] | Phase 1, healthy subjects (obese and normal weigh) (n=24) | Linear, 2-compartment (body weight) |

a. Number of subjects/patients denotes the number of study participants who received at least one dose of posaconazole, not the total number of participants in the study.

**Table S6. Posaconazole publications for model qualification**

| Citation | Patient population | Dose |
|---|---|---|
| Cornely *et al*. J Antimicrob Chemother 2017;72:3406-3413. [4] | Patients at high risk of invasive fungal infections (n=237) | 300 mg IV BID on Day 1 followed by 300 mg IV QD for 10 days; could switch to PO thereafter |
| Duarte *et al*. Antimicrob Agents Chemother 2014;58(10):5758-5765. [2] | Patients with MDS or AML (n=54) | 200 or 300 mg using standard load (BID on Day 1) and maintenance (QD); tablet formulation |
| Dvorackova *et al*. Antibiotics 2023;12 (9):1399. [9] | Adult lung transplant patients receiving posaconazole as either prophylaxis or treatment (n=32) | 300 mg PO QD then adjusted (100-400 mg QD) based on therapeutic drug monitoring; tablet formulation |
| Liu *et al*. Adv Ther 2020;37:2493-2506. [6] | Chinese patients with AML (n=65) | 300 mg PO BID on Day 1 followed by 300 mg PO QD; tablet formulation |
| Petitcollin *et al*. Antimicrob Agents Chemother 2017;61e0116617. [3] | Hospitalized patients with hematologic malignancies (n=49) | 300 mg PO BID on Day 1 followed by 300 mg PO QD; tablet formulation |
| Sime *et al*. Antimicrob Agents Chemother 2018;62(6):e00242-18. [5] | ICU patients with suspected or confirmed fungal infection (n=8) | 300 mg IV single dose |

| | | |
|---|---|---|
| Wasmann *et al*. J Antimicrob Chemother 2020;75:1006-1013. [7] | Phase 1, healthy subjects (obese vs normal weight patients, n=24) | Normal weight: 300 mg IV single dose<br>Obese: 300 or 400 mg IV single dose |
| Wu *et al*. Adv Ther 2022;39:1697-1710. [8] | Asian adults with MDS or AML (n=70) | 300 mg PO BID on Day 1 followed by 300 mg PO QD; tablet formulation |

Note: AML, acute myelogenous leukemia; BID, twice daily; MDS, myelodysplastic syndrome; mg, milligrams; n, number of subjects/patients; PO, by mouth; QD, once daily.

**Figure S1. Qualification of Iwasa posaconazole model with: PK data from Duarte *et al.* (2014) [2]**

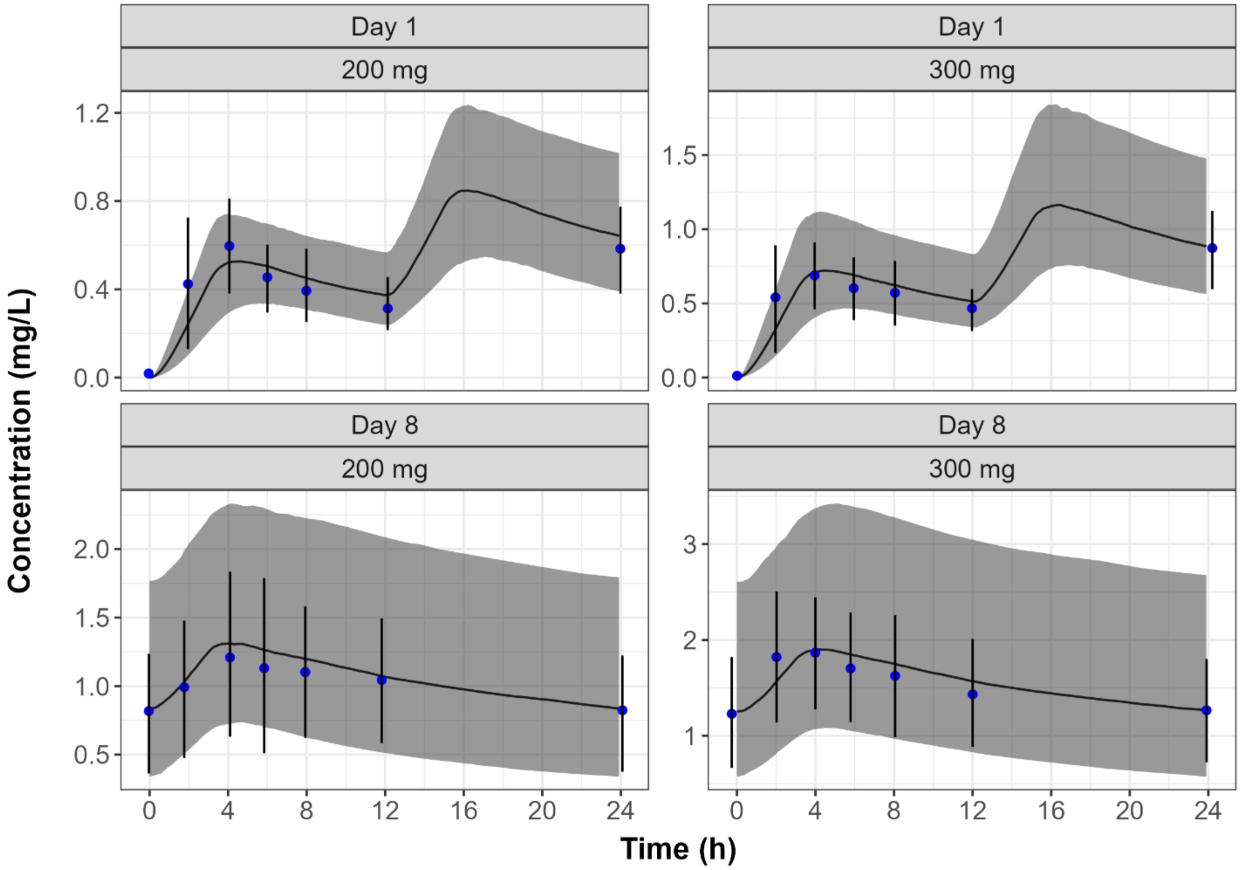


Blue dots and error bars show observed mean (+/-SD) concentrations digitized from publication (Figure 2)
Black line and shaded region represent the median and 90% prediction interval from model-based simulations.

Note: h, hours; L, liters; mg, milligrams; SD, standard deviation.

**Figure S2. Qualification of Iwasa posaconazole model with: PK data from Petitcollin *et al*. (2017) [3]**

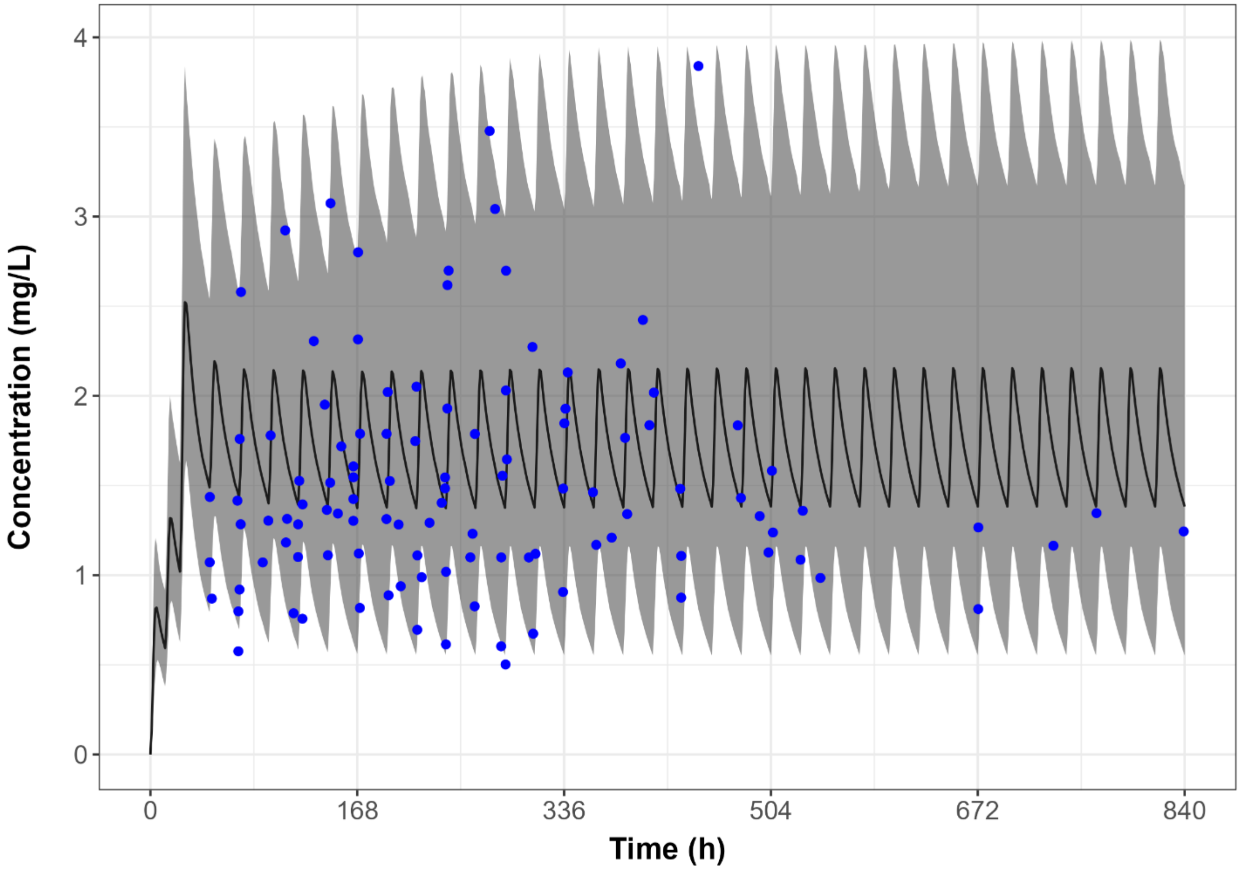


Blue dots are observed concentrations digitized from publication (Figure 2)
Black line and shaded region represent the median and 90% prediction interval from model-based simulations.

Note: h, hours; L, liters; mg, milligrams.

**Figure S3. Qualification of Iwasa posaconazole model with: PK data from Cornely *et al*. (2017) [4]**

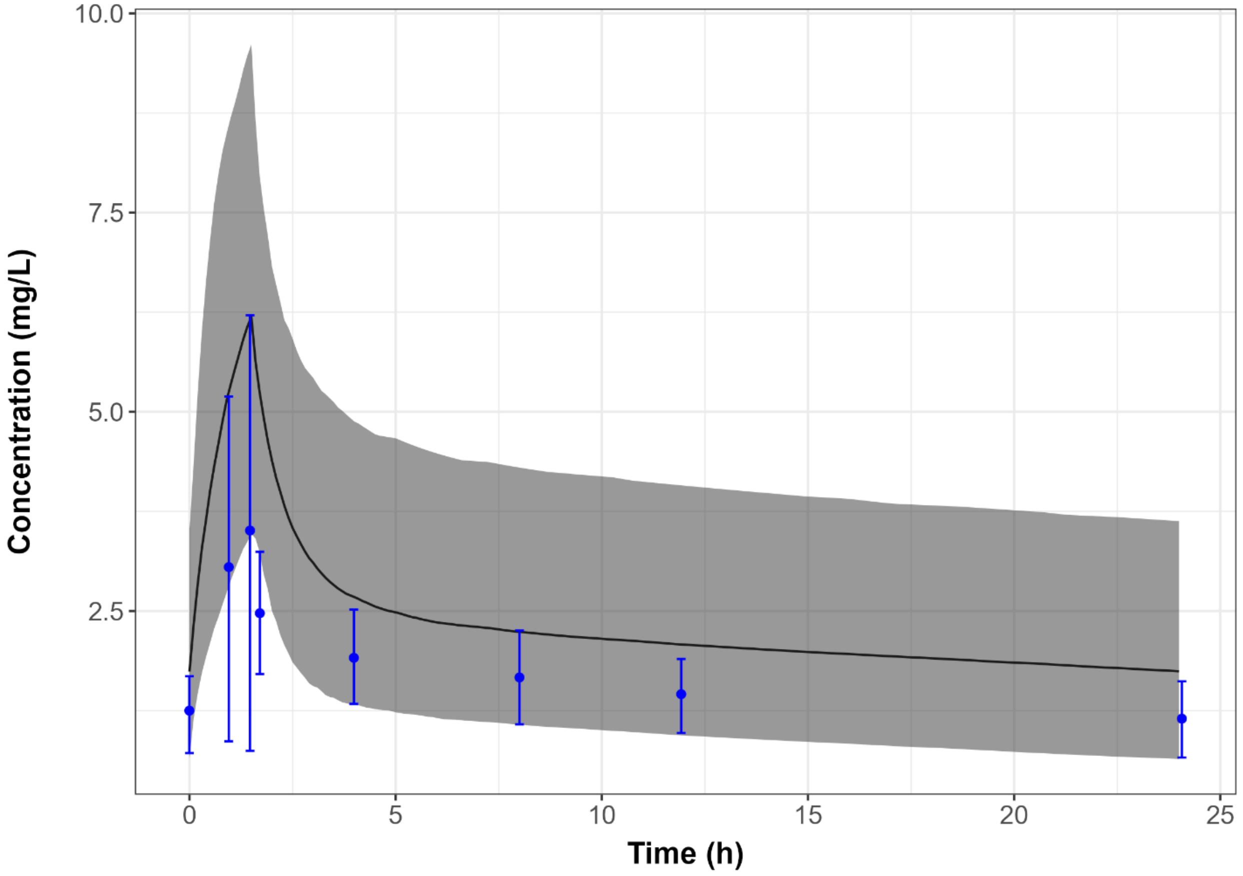


Blue dots and error bars show observed mean (+/-SD) concentrations digitized from publication (Figure 2)
Black line and shaded region represent the median and 90% prediction interval from model-based simulations.

Note: h, hours; L, liters; mg, milligrams; SD, standard deviation.

**Figure S4. Qualification of Iwasa posaconazole model with: PK data from Sime *et al*. (2018) [5]**

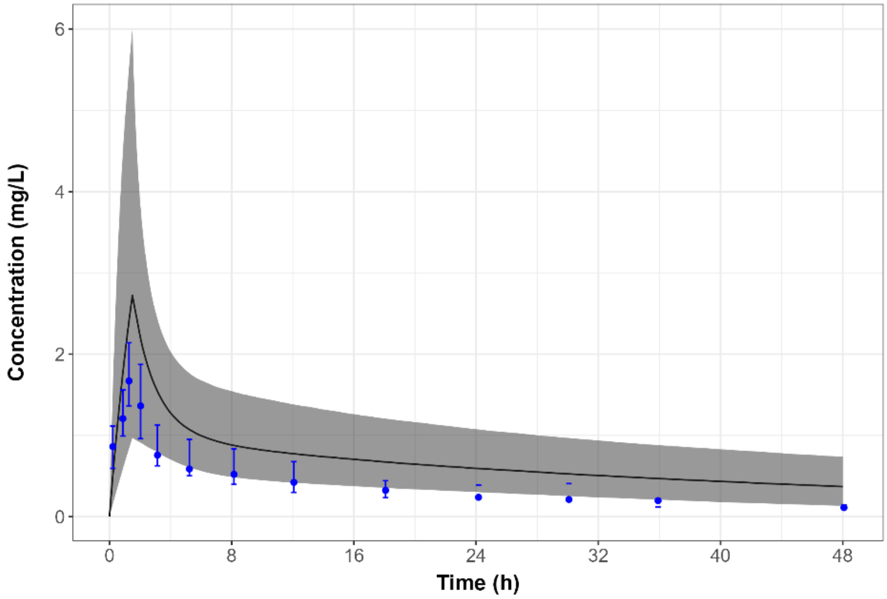


Blue dots and error bars show observed median (IQR) concentrations digitized from publication (Figure 1).
Note that some IQR not fully digitizable
Black line and shaded region represent the median and 90% prediction interval from model-based simulations.

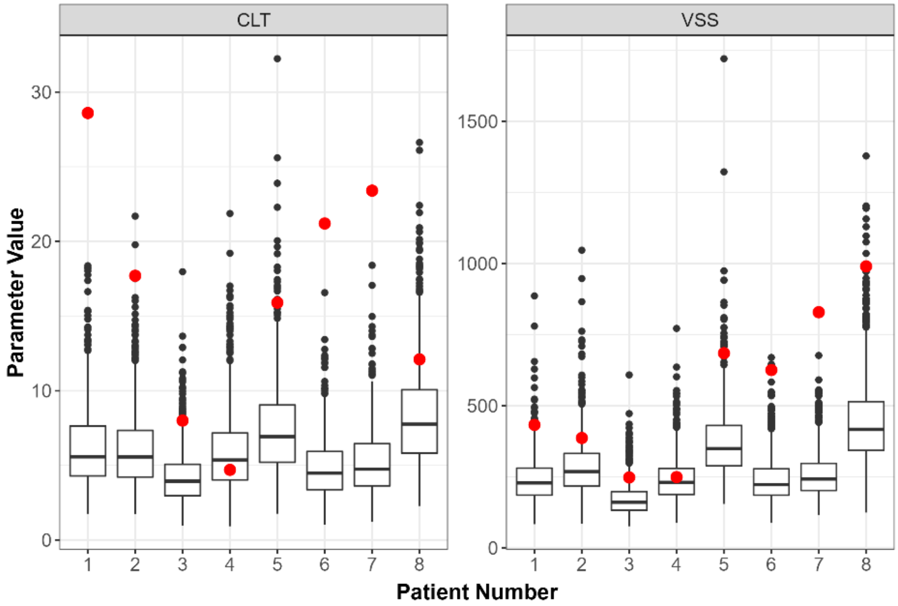


Red dots are individual PK parameter estimates reported in Table 2 of the publication.
Box and whisker plots show distribution of predicted parameters for each patient
based on 1000 replicates of the patient demographics.

Note: CLT, total clearance; h, hours; IQR, interquartile range; L, liters; mg, milligrams; VSS, volume of distribution at steady state.

**Figure S5. Qualification of Iwasa posaconazole model with: PK data from Liu *et al.* (2020) [6]**

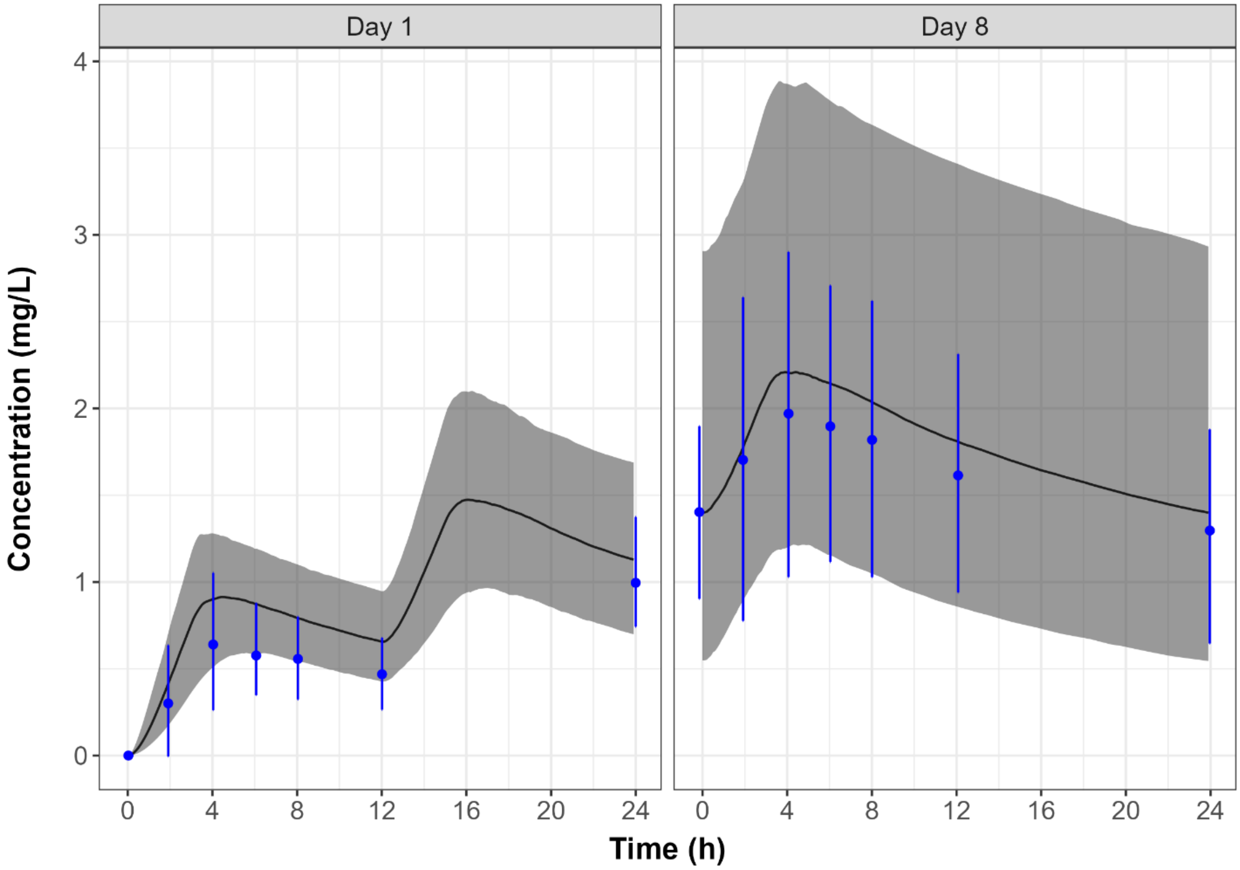


Blue dots and error bars show observed mean (+/-SD) concentrations digitized from publication (Figure 1)
Black line and shaded region represent the median and 90% prediction interval from model-based simulations.

Note: h, hours; L, liters; mg, milligrams; SD, standard deviation.

**Figure S6. Qualification of Iwasa posaconazole model with: PK data from Wasmann *et al*. (2020) [7]**

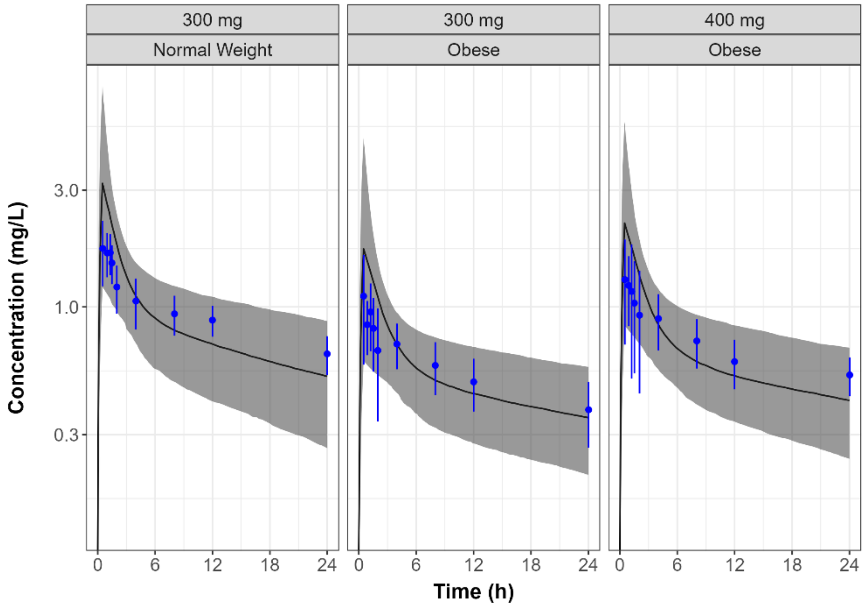


Blue dots and error bars show observed mean (+/-SD) concentrations digitized from publication (Figure 1)
Black line and shaded region represent the median and 90% prediction interval from model-based simulations.

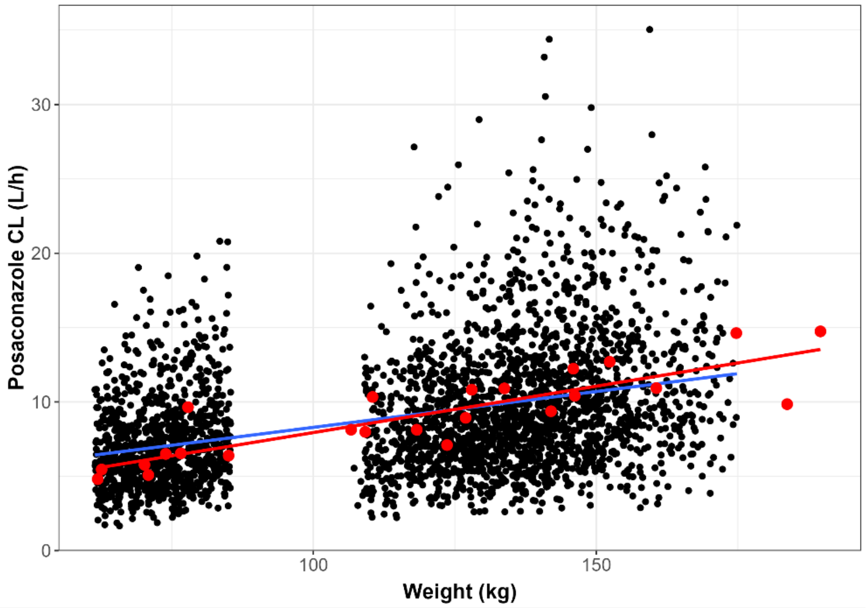


Red dots show reported CL values (Figure 2a) with red line showing linear smoother through reported values.
Black dots are simulated CL values with blue line showing linear smoother through the simulated values.

Note: CL, clearance; h, hours; kg, kilograms; L, liters; mg, milligrams; SD, standard deviation.

**Figure S7. Qualification of Iwasa posaconazole model with: PK data from Wu *et al*. (2022) [8]**

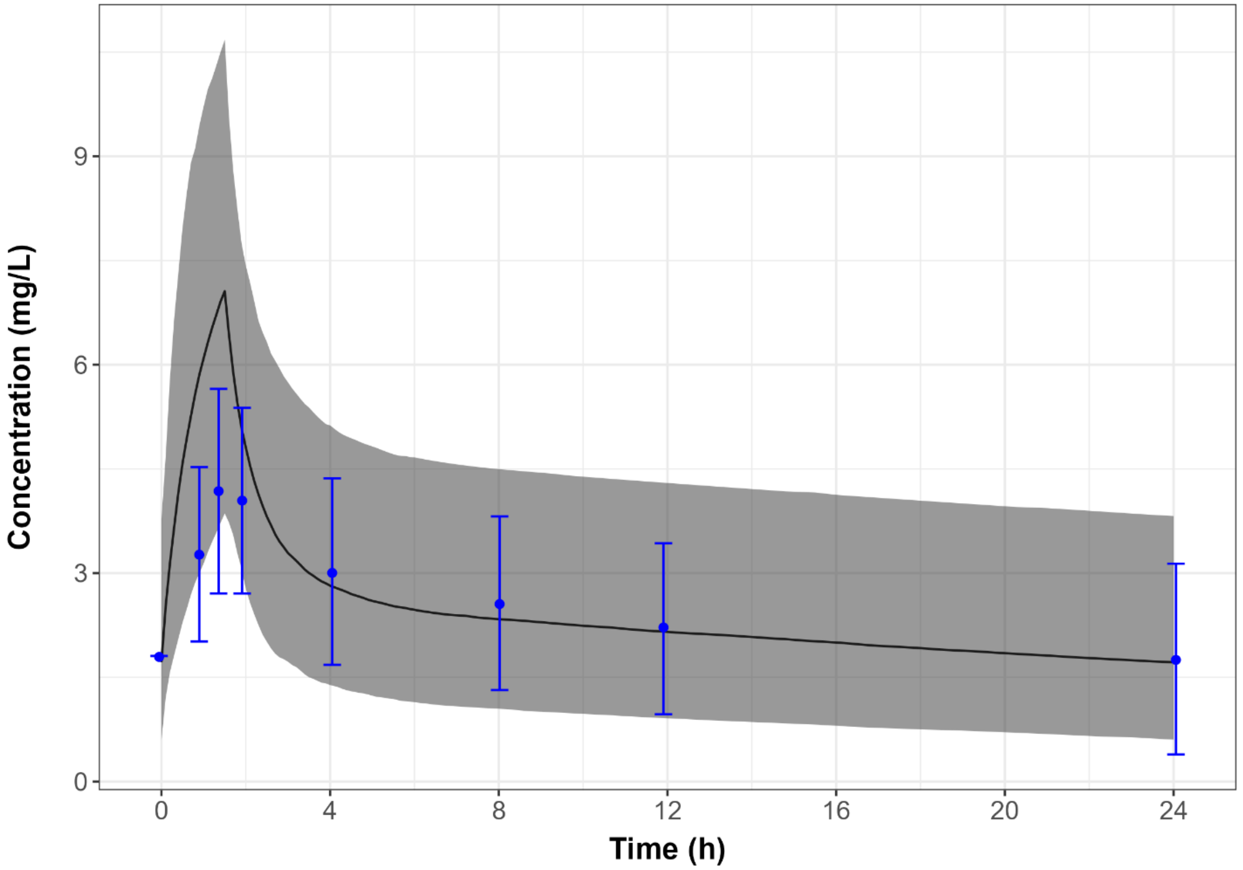


Blue dots and error bars show observed mean (+/-SD) concentrations digitized from publication (Figure 1)
Black line and shaded region represent the median and 90% prediction interval from model-based simulations.

Note: h, hours; L, liters; mg, milligrams; SD, standard deviation.

**Figure S8. Qualification of Iwasa posaconazole model with: PK data from Dvorackova *et al*. (2023) [9]**

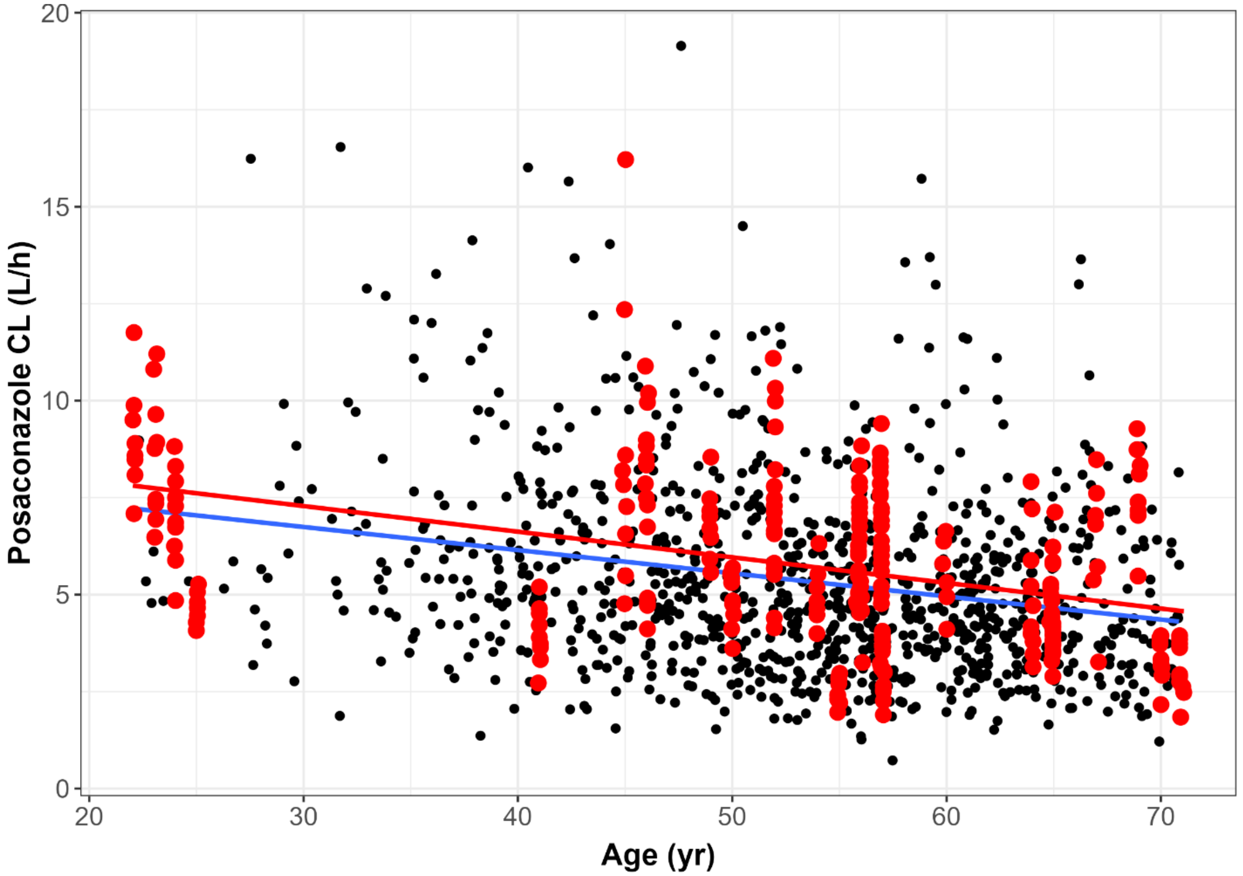


Red dots show reported CL values (Figure 1) with red line showing linear smoother through reported values.
Black dots are simulated CL values with blue line showing linear smoother through the simulated values.

Note: CL, clearance; h, hours; L, liters; yr, years.

## References

1. Iwasa T, de Almeida C, Fauchet F, *et al*. Model-informed dose justifications for posaconazole in Japanese patients for prophylaxis and treatment against fungal infection. J Clin Pharmacol 2023;63(4):421-434.
2. Duarte RF, López-Jiménez J, Cornely OA, et al. Phase 1b study of new posaconazole tablet for prevention of invasive fungal infections in high-risk patients with neutropenia. Antimicrob Agents Chemother 2014;58(10):5758-5765.
3. Petitcollin A, Boglione-Kerrien C, Tron C, et al. Population pharmacokinetics of posaconazole tablets and Monte Carlo simulations to determine whether all patients should receive the same dose. Antimicrob Agents Chemother 2017;61(11):e01166-17.
4. Cornely OA, Robertson MN, Haider S, et al. Pharmacokinetics and safety results from the Phase 3 randomized, open-label, study of intravenous posaconazole in patients at risk of invasive fungal disease. J Antimicrob Chemother 2017;72(12):3406-3413. Erratum in: J Antimicrob Chemother 2017;72(12):3501.
5. Sime FB, Stuart J, Butler J, et al. Pharmacokinetics of intravenous posaconazole in critically ill patients. Antimicrob Agents Chemother 2018;62(6):e00242-18.
6. Liu K, Wu D, Li J, et al. Pharmacokinetics and safety of posaconazole tablet formulation in Chinese participants at high risk for invasive fungal infection. Adv Ther 2020;37(5):2493-2506.
7. Wasmann RE, Smit C, van Donselaar MH, et al. Implications for IV posaconazole dosing in the era of obesity. J Antimicrob Chemother 2020;75(4):1006-1013.
8. Wu D, Mi Y, Weng J, et al. Phase 1b/3 pharmacokinetics and safety study of intravenous posaconazole in adult Asian participants at high risk for invasive fungal infections. Adv Ther 2022;39(4):1697-1710.

9. Dvořáčková E, Šíma M, Zajacová A, et al. Dosing optimization of posaconazole in lung-transplant recipients based on population pharmacokinetic model. Antibiotics (Basel) 2023;12(9):1399.
10. Jansen AME, Muilwijk EW, van der Velden WJFM, et al. Posaconazole bioavailability of the solid oral tablet is reduced during severe intestinal mucositis. Clin Microbiol Infect 2022;28(7):1003-1009.
11. Pena-Lorenzo D, Rebollo N, Sanchez-Hernandez J G, et al. Population pharmacokinetics of a posaconazole tablet formulation in transplant adult allogeneic stem cell recipients. Eur J Pharm Sci 2022;168:106049.
12. van Iersel MLPS, Rossenu S, de Greef R, Waskin H. A population pharmacokinetic model for a solid oral tablet formulation of posaconazole. Antimicrob Agents Chemother 2018;62:e02465-17.
